# Conservation, abundance, glycosylation profile, and localization of the TSP protein family in *Cryptosporidium parvum*

**DOI:** 10.1016/j.jbc.2023.103006

**Published:** 2023-02-10

**Authors:** Alan John, Stefanie M. Bader, Niccolay Madiedo Soler, Kharizta Wiradiputri, Swapnil Tichkule, Sean T. Smyth, Stuart A. Ralph, Aaron R. Jex, Nichollas E. Scott, Christopher J. Tonkin, Ethan D. Goddard-Borger

**Affiliations:** 1 The Walter and Eliza Hall Institute of Medical Research, Parkville, Victoria, Australia; 2 Department of Medical Biology, University of Melbourne, Parkville, Victoria, Australia; 3 Department of Biochemistry and Pharmacology, Bio21 Molecular Science and Biotechnology Institute, University of Melbourne, Parkville, Victoria, Australia; 4 Department of Microbiology and Immunology, University of Melbourne at the Peter Doherty Institute for Infection and Immunity, Parkville, Victoria, Australia

**Keywords:** *Cryptosporidium parvum*, cryptosporidiosis, thrombospondin repeat, vaccine, proteomics, glycoproteomics, protein glycosylation, expansion microscopy, apicomplexa, tryptophan C-mannosylation, TSR

## Abstract

*Cryptosporidium parvum* is a zoonotic apicomplexan parasite and a common cause of diarrheal disease worldwide. The development of vaccines to prevent or limit infection remains an important goal for tackling cryptosporidiosis. At present, the only approved vaccine against any apicomplexan parasite targets a conserved adhesin possessing a thrombospondin repeat domain. *C. parvum* possesses 12 orthologous thrombospondin repeat domain–containing proteins known as *Cp*TSP1–12, though little is known about these potentially important antigens. Here, we explore the architecture and conservation of the *Cp*TSP protein family, as well as their abundance at the protein level within the sporozoite stage of the life cycle. We examine the glycosylation states of these proteins using a combination of glycopeptide enrichment techniques to demonstrate that these proteins are modified with C-, O-, and N-linked glycans. Using expansion microscopy, and an antibody against the C-linked mannose that is unique to the *Cp*TSP protein family within *C. parvum*, we show that these proteins are found both on the cell surface and in structures that resemble the secretory pathway of *C. parvum* sporozoites. Finally, we generated a polyclonal antibody against *Cp*TSP1 to show that it is found at the cell surface and within micronemes, in a pattern reminiscent of other apicomplexan motility–associated adhesins, and is present both in sporozoites and meronts. This work sheds new light on an understudied family of *C. parvum* proteins that are likely to be important to both parasite biology and the development of vaccines against cryptosporidiosis.

Diarrheal diseases are the third leading cause of death in children under 5 years of age, with those in the developing world being at greatest risk (1, 2). The most prevalent etiological agents responsible for severe diarrhea in children are rotavirus, *Cryptosporidium* spp., enterotoxigenic *Escherichia coli*, and *Shigella* spp (3, 4). Interventions targeting these pathogens, especially vaccines, have the potential to substantially reduce childhood morbidity and mortality. Indeed, the rotavirus vaccines, which protect against the leading cause of childhood diarrheal disease, have decreased deaths associated with acute gastroenteritis in children under 5 years of age by 36% (5). Comparable progress has yet to be realized against other diarrheal diseases, including cryptosporidiosis, which is the second-leading cause of moderate-to-severe diarrheal disease in children (3).

Whilst cryptosporidiosis is usually acute and self-limiting in immunocompetent individuals, chronic infection can occur in malnourished and/or immunocompromised individuals, especially those with a poorly managed HIV infection. Such chronic infections in children can impair physical and cognitive development (6). Treatment options are limited: nitazoxanide is the only Food and Drug Administration–approved drug for this disease, and it has poor efficacy in immunocompromised patients: the cohort that most needs therapeutic intervention (7).

Currently, no vaccine is available for the prevention of cryptosporidiosis, although such a product is plausible given that prior infections confer resistance to subsequent infection (8, 9). Studies in AIDS patient populations have demonstrated the importance of T-cell responses in controlling *Cryptosporidium* spp. (10), although mucosal immunoglobulin A responses also play a role in limiting cryptosporidiosis (11, 12). A vaccine capable of eliciting similar mucosal immune responses could be invaluable in combating severe diarrheal diseases. However, identifying antigens that provide broad protection against the various *Cryptosporidium* spp. responsible for disease in humans is a major development hurdle. *Cryptosporidium parvum* and *Cryptosporidium hominis* are the two species most frequently responsible for disease in humans. Identifying conserved and secreted antigens in these parasites represents an important step toward rationally designed vaccines.

Among the many *Cryptosporidium* proteins that might serve as vaccine antigens, those with a thrombospondin repeat (TSR) domain are of particular interest, because of the importance of similar proteins in the life cycle of related apicomplexan parasites like the *Plasmodium* spp. and *Toxoplasma gondii*. In these parasites, secreted TSR-containing proteins, which are commonly type I or II transmembrane adhesins, are involved in parasite motility, host cell invasion, traversal, and egress (13, 14, 15, 16, 17, 18). The only malaria vaccine that is presently approved, GSK’s RTS,S/AS01, uses the *Plasmodium falciparum* TSR-containing circumsporozoite protein as antigen, thereby elevating this family of proteins as privileged vaccine antigens (19). In *Cryptosporidium* spp., there are 12 well-conserved syntenic genes encoding TSR-containing proteins: in *C. parvum*, these are denoted *Cp*TSP1–12 (Fig. 1*A*). Surprisingly, little is known about this family of proteins.

**Figure 1:**
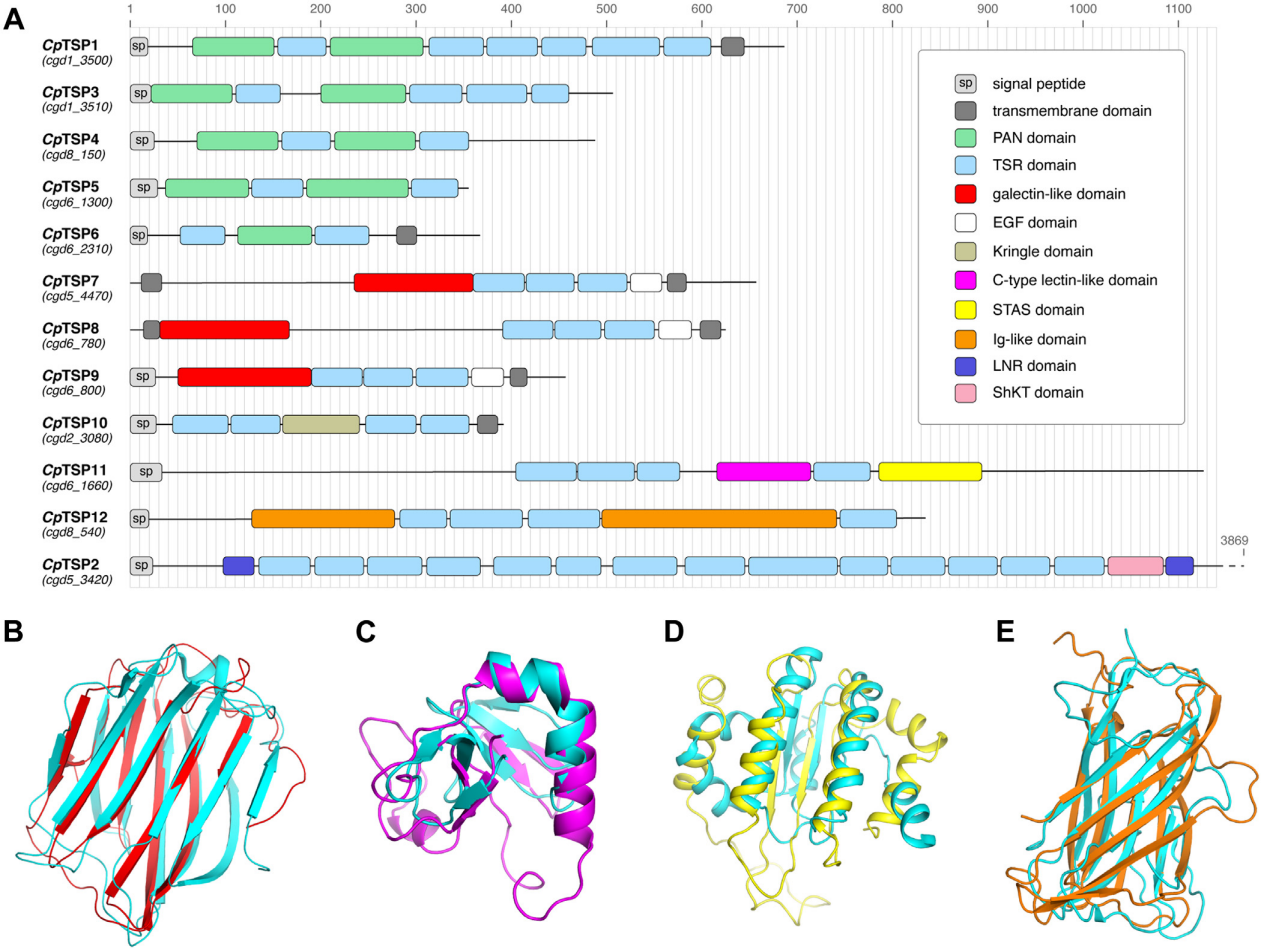
**Architecture of the *Cryptosporidium parvum* TSR proteins.***A*, domain architecture of the *C. parvum* TSR proteins *Cp*TSP1–12, as determined using structural models built using AlphaFold 2. The corresponding genes are provided in *parentheses*. *B*, superposition of galectin-like domains from human galectin-4 (Protein Data Bank [PDB] ID: 5DUV, *cyan*) and *Cp*TSP7 model (*red*). *C*, superposition of the C-type lectin domains from *Polyandrocarpa misakiensis* TC14 (PDB ID: 1BYF, *cyan*) and *Cp*TSP11 model (*magenta*). *D*, superposition of STAS domains from *Bacillus subtilis* YtvA (PDB ID: 2MWG, *cyan*) and *Cp*TSP11 model (*yellow*). *E*, superposition of immunoglobulin (Ig)-like domains in *Corynebacterium diphtheriae* membrane protein (PDB ID: 3LSO, *cyan*) and *Cp*TSP12 model (*orange*). TSR, thrombospondin repeat.

Here, we begin to address the paucity of information available for the *Cp*TSP protein family. We revisited the domain architecture and inter-relatedness of these proteins using the AlphaFold2 (20) algorithm to predict domain boundaries within these proteins, providing insights into what domains are unique and conserved across the family. Using a population genetics approach, we quantitated how conserved each of the TSR proteins are within the *C. parvum* population and gained insights into the evolutionary pressures on each potential antigen. Global proteomic profiling of excysted sporozoites then demonstrated that *Cp*TSP1-4 and *Cp*TSP7-12 are present at the protein level in *C. parvum* sporozoites, and that these proteins are modified with C-, O-, and N-linked glycans. Finally, expansion microscopy and standard immunofluorescent imaging employing a monoclonal antibody directed against unique glycosylation on TSR proteins, as well as an affinity-purified polyclonal antibody to *Cp*TSP1, confirmed the localization of the TSR proteins, and *Cp*TSP1 specifically, to the sporozoite secretory pathway and cell surface. Collectively, these data provide important insights into these largely uncharacterized proteins and serve as an important foundation for future study and development of the *Cp*TSP proteins as vaccine antigens.

## Results

### Domain architecture of the *C. parvum* TSR proteins

The initial description of the TSR proteins in *C. parvum* used exhaustive sequence alignment analyses to identify the modular domains that comprise these 12 proteins (21). These published domain assignments differed in many instances to those captured by the InterPro database (22). Furthermore, both the original description and aforementioned database failed to identify domains in large regions of *Cp*TSP7–9 and *Cp*TSP11–12, despite those regions having appreciable predicted secondary structure elements. To address these issues, we revisited the domain architecture problem by building structural models of *Cp*TSP1–12 using AlphaFold 2 (20) and manually assigned the boundaries of each domain. This approach revealed the presence of hitherto unrecognized domains, which were classified using structural homology searches with DALI (Table S1) (23). This provided a more complete map of *C. parvum* TSR protein architecture (Fig. 1*A*). While it largely mirrors the original description (21), it builds on this work through the addition of TSR domains in *Cp*TSP2, *Cp*TSP6, *Cp*TSP11, and *Cp*TSP12; galectin-like domains in *Cp*TSP7–9 (Fig. 1*B*); C-type lectin (Fig. 1*C*) and STAS (Fig. 1*D*) domains in *Cp*TSP11; and immunoglobulin (Ig)-like domains in *Cp*TSP12 (Fig. 1*E*). The identification of galectin-like and C-type lectin-like domains allude to a possible function for *Cp*TSP7–9 and *Cp*TSP11 as secreted adhesins that bind host glycoproteins. In a similar fashion, the PAN domains of *Cp*TSP1,3–6 share similarities with the galactose-binding PAN domains from *T. gondii* MIC4 (24) and *Sarcocystis muris* SML2 (25), suggesting that the former proteins may also be lectins involved in adhesion to the host cell glycocalyx or to mucus itself. Domain architectural similarities among *Cp*TSP1,3–5 and *Cp*TSP7–9 are suggestive of similar or even redundant functions, although this remains to be demonstrated.

### Diversity of the *C. parvum* TSR proteins

Understanding the population-level diversity of the *Cp*TSP proteins and their expression levels in different stages of the parasite’s life cycle is important for determining their appropriateness as vaccine antigen candidates. We compared genomes from 32 *C. parvum* isolates to obtain population genetic indices (nucleotide diversity, number of segregating sites, and Tajima’s D values) for the genes encoding the 12 TSR-containing proteins (Table S2).

Tajima’s D is a population genetics statistical test used to determine if a gene is evolving neutrally (Tajima’s D = 0), under purifying selection (Tajima’s D < 0), or under balancing selection (Tajima’s D > 0). With the exception of the gene encoding *Cp*TSP12, most genes encoding *Cp*TSP proteins are under purifying selection (Table S2), which means that deleterious mutations are being selectively removed from this population. This implies that these genes contribute to parasite fitness.

**Figure 2:**
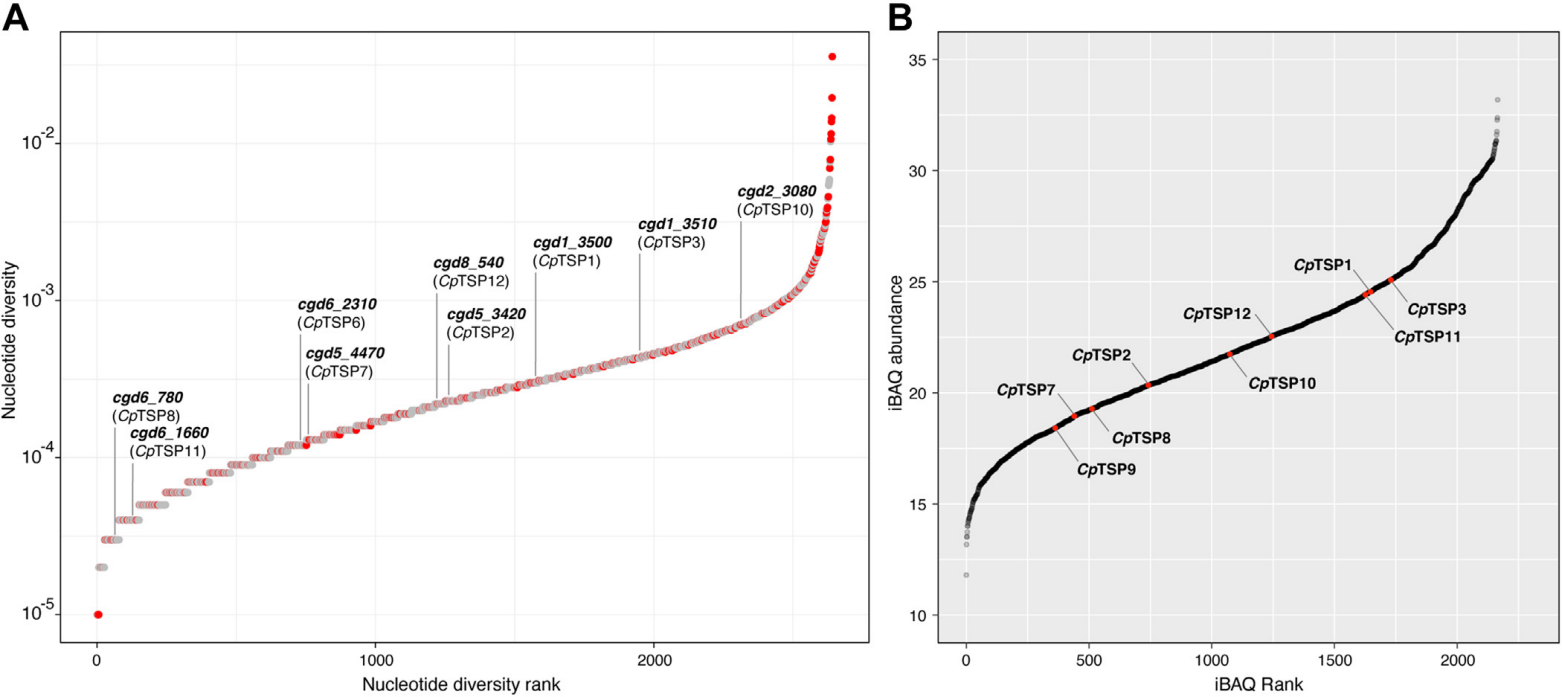
**Diversity of the *Cryptosporidium parvum* TSR proteins and their abundance in sporozoites.***A*, the 2641 polymorphic *C. parvum* genes (nucleotide diversity >0) ranked with respect to their nucleotide diversity. Genes encoding proteins with a putative signal peptide are indicated in *red* and those encoding *C. parvum* TSR proteins are annotated, with the corresponding protein name in *parentheses*. *B*, the *C. parvum* sporozoite proteome ranked by iBAQ values with observed TSR proteins indicated in *red* and annotated. iBAQ, intensity-based absolute quantification; TSR, thrombospondin repeat.

To provide some context for how polymorphic these genes are relative to the rest of the genome, we ranked and plotted the nucleotide diversity of the 68% of *C. parvum* genes that are polymorphic (nucleotide diversity >0) and annotated where the genes encoding *Cp*TSP proteins sit within this hierarchy (Fig. 2*A*). Genes encoding *Cp*TSP4,5,9 are not polymorphic and while the other *Cp*TSP genes have varying degrees of polymorphism, none could be regarded as highly polymorphic: they are all well conserved among *C. parvum* isolates. This is commensurate with the relatively small number of segregating sites (polymorphic nucleotide positions) within these genes.

Building on this further, we compared the *C. parvum* TSR proteins to their orthologs in *C. hominis*, another human pathogen. The *C. hominis* orthologs share >90% amino acid identity, with the exception of TSP8, which has 86.9% identity because of a frame shift mutation in *C. parvum* (26). Collectively, these data indicate that most, if not all, of these TSR proteins are sufficiently conserved and relevant to *C. parvum* fitness to serve as vaccine antigen candidates. It also suggests that *C. parvum* antigens are likely to elicit substantial crossreaction with orthologous *C. hominis* antigens.

### Abundance of the TSR proteins in *C. parvum* sporozoites

To probe the protein-level abundance of the *Cp*TSP proteins, we turned to proteomic studies of *C. parvum* sporozoites, which were obtained en masse by excystation of commercially available oocysts. These were lyzed with SDS, and the protein extract was digested with trypsin prior to analysis by LC–MS/MS with field asymmetric waveform ion mobility spectrometry (FAIMS)–based fractionation (27, 28). Peptides from nine of the 12 TSR proteins were detected. Their relative abundance, as determined by ranked intensity-based absolute quantification values (29), varied significantly: *Cp*TSP1,3,11 expression levels were in the top quartile of the sporozoite proteome; *Cp*TSP2,10,12 were in the middle two quartiles; and *Cp*TSP7–9 were in the bottom quartile (Fig. 2*B*).

### Glycosylation of TSR proteins in *C. parvum* sporozoites

**Figure 3:**
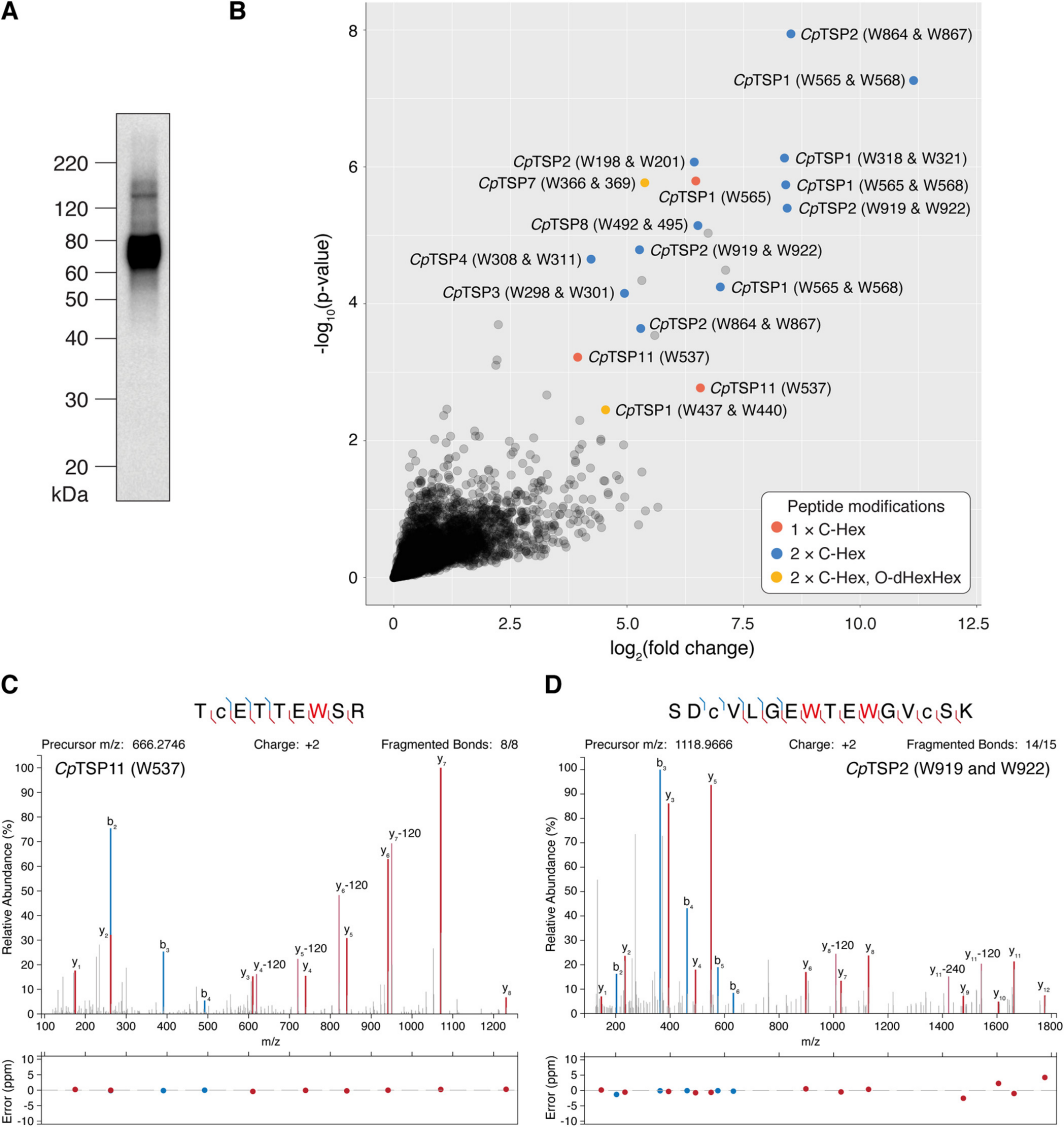
**C-mannosylated proteins in *Cryptosporidium parvum* sporozoites.***A*, Western blot analysis of SDS-extracted proteins from *C. parvum* sporozoites using the 5G12 mAb as primary antibody. *B*, volcano plot demonstrating the enrichment of C-mannosylated peptides from *C. parvum* sporozoite lysate using the 5G12 mAb, as compared with an isotype control. *C*, tandem mass spectra for the singly modified *Cp*TSP11 peptide ^531^TCETTEWSR^539^ and (*D*) for the doubly modified *Cp*TSP2 peptide ^912^SDCVLGEWTEWGVCSK^927^. A *red* “W” indicates a Trp(Man) residue, whereas “c” represents acetamidylcysteine.

The same sporozoite peptide samples were then subjected to two different glycopeptide-enrichment strategies and LC–MS/MS analysis to determine the native glycosylation states of these and other *C. parvum* proteins, since this information is useful in the design of vaccine antigens (30). The first enrichment strategy made use of the 5G12 antibody, which was developed to recognize peptides bearing the C-mannosyl tryptophan protein modification (31, 32). This unusual type of glycosylation, as well as O-linked glucosyl-β(1→3)-fucosylation [βGlc (1→3)αFuc], is commonly associated with TSR domains in metazoans (33) and apicomplexans like *Plasmodium* spp (34, 35) and *T. gondii* (36, 37). We suspected that these modifications existed in *Cryptosporidium* spp. too, since genes encoding the putative enzymes that install these modifications are conserved and syntenic: for *C. parvum*, these include the tryptophan C-mannosyltransferase “dpy-19” (*cgd4_2180*), protein O-fucosyltransferase “pofut2” (*cgd1_2440*), and glucosyltransferase “b3glct” (*cgd5_540*). Western blot analysis of *C. parvum* sporozoite lysate using the 5G12 monoclonal antibody (mAb) confirmed the presence of many proteins bearing C-mannosyl tryptophan (Figs. 3*A* and S1). The broadness of the band around 60 to 80 kDa reflects the fact that several *Cp*TSP proteins have sizes in this range and are likely present as a heterogenous mixture of glycoforms. To determine which proteins possessed the modifications, immunoprecipitations were performed in quintuplicate on the trypsin-digested lysate using either the 5G12 mAb or an isotype control. These samples were analyzed by LC–MS/MS, and data were searched for peptides modified with Trp(Hex), Ser/Thr(dHex), and Ser/Thr(dHexHex), which are commonly found in proximity to each other on TSR domains (38). Seventeen highly enriched modified peptides decorated with combinations of Trp(Hex) and Ser/Thr(dHexHex) across multiple TSR domains from *Cp*TSP1–4,7–8,11 were identified (Fig. 3, *B*–*D* and Table 1). No peptides with Ser/Thr(dHex) were observed in this data set, suggesting that glucosylation of O-linked fucose is an efficient process in *C. parvum* sporozoites. Manual inspection of data generated from these glycopeptides revealed the characteristic 120 Da loss associated with fragmentation of the C-glycoside in Trp(Hex) residues, enabling assignment of the C-mannosylation sites with a high degree of confidence (Fig. 3, *C* and *D*). Due to the use of higher energy collision dissociation (HCD) fragmentation, the sites of the more labile dHexHex modifications could not be determined beyond the characteristic loss of dHexHex from the peptide backbone (Fig. S2). It is likely that this glycan is localized to the classical CXX(**S/T)**S motif of the TSR domain, as it is in other apicomplexans (34, 35, 36, 37) and metazoans (33). These data confirm that C-mannosylation occurs in *C. parvum* sporozoites and that it is only found on TSR proteins, at least in this stage of the life cycle.

**Table 1:** N-, O-, and C-glycosylation sites identified on *Cp*TSP proteins in *Cryptosporidium parvum* sporozoites following 5G12 or ZIC-HILIC enrichment

Protein	UniProt ID	Peptide	No. of glycosylation sites		
			W-Hex	S/T-dHexHex	N-Hex_5–6_ HexNAc_2_
*Cp*TSP1 (TRAP-C1)	Q5CSA5	YTE**W**SA**W**SSCDCSGTQTR	2	—	—
		DADCDTGTCIHNE**W**SS**W**TTCKDPCSNTETMSR	2	—	—
		ESCNKDVECPHVQCELGE**W**SS**W**SPCSV**T**CGCGTTTR	2	1	—
*Cp*TSP2	Q5CRC0	SDCVLGE**W**TE**W**GVCSK	2	—	—
		NGGETCGALKAEETGCNSHIPCPLSCTVSE**W**GN**W**SR	2	—	—
		VGE**W**SS**W**SECDAK	2	—	—
*Cp*TSP3	Q5CSA4	YGECDINCVLGD**W**TQ**W**SGCDSALCSDGK	2	—	—
*Cp*TSP4	Q5CQ00	CFVGE**W**SN**W**SK	2	—	—
*Cp*TSP7	Q5CQ18	VEDCQISQ**W**TD**W**STCSK**T**CSTGSK	2	1	—
*Cp*TSP8 (*Cp*MIC1)	Q5CXK1	ELTHSAPGCDSLLKETSSCNSSPCPVDCVLSF**W**SP**W**TGCSK	2	—	—
		YYFDDKNLYYV**N**STGIDEK	—	—	1
*Cp*TSP11	Q5CXC2	TCETTE**W**SR	1	—	—
		LSSIK**N**ETEQ**N**SSIQTGDLLTK	—	—	1
		ELKFNGL**N**ITSYENR	—	—	1

Bold and underlined residues are confirmed glycosylation sites, whereas bold-only residues are likely but unconfirmed sites of O-glycosylation.

**Figure 4:**
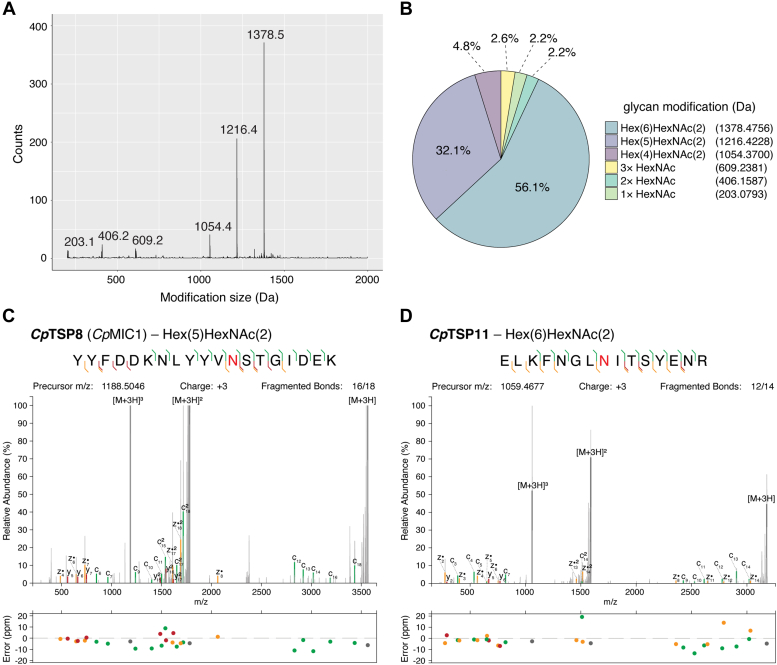
**O- and N-glycosylation in *Cryptosporidium parvum* sporozoites.***A*, the open search plot for glycans within HILIC-enriched glycopeptides from *C. parvum* sporozoites. *B*, the relative proportions of each glycan modification within HILIC-enriched glycopeptides from *C. parvum* sporozoites. *C* and *D*, tandem mass spectra of representative N-glycosylated peptides from *Cp*TSP proteins providing localization information (the site of modification is indicated by *red text*). HILIC, hydrophilic interaction liquid chromatography.

While no N-glycan data are presently available for the *Cp*TSP family proteins, a previous glycoproteomic study on *C. parvum* revealed that minimally processed Hex_5–6_HexNAc_2_ structures predominate in this organism, and that they are mainly found on the NXT sequon (39). However, this prior work identified just 32 glycopeptides across 16 unique proteins (39), none of which were from the *Cp*TSP protein family. To obtain a richer data set, and coverage of the *Cp*TSPs, we enriched glycopeptides from trypsin-digested *C. parvum* sporozoite lysate using zwitterionic-hydrophilic interaction liquid chromatography (ZIC–HILIC). Analysis of this sample using HCD and electron-transfer hcd (EThcD) on a Orbitrap Lumos, followed by open database searching using MSFragger, provided over 1000 unique peptide-spectrum matches corresponding to 286 unique glycopeptide sequences. Open searching enabled the identification of glycopeptides in an unbiased manner: no constraints based on assumptions of glycan structure were made (40). This confirmed that peptides with N-linked Hex_4–6_HexNAc_2_ structures comprised around 90% of all identified *C. parvum* sporozoite peptide-spectrum matches with a mass greater than 200 Da, with the remainder being peptides with 1 to 3 HexNAc units (Fig. 4, *A* and *B*). Manual assignment of EThcD spectra for glycopeptides with multiple HexNAc units revealed that each HexNAc is connected to a different residue (Fig. S3 and Data S1). Given previous reports of mucin-type glycosylation in *C. parvum*, these O-glycans are likely to be Tn structures (GalNAcα1-Ser/Thr) (41, 42).

For the *Cp*TSP family of proteins, multiple glycopeptides were identified (Table 1) with EThcD analysis confirming that these delta masses corresponded to N-glycosylation events with both Hex_5_HexNAc_2_ and Hex_6_HexNAc_2_ localized to the peptide ^212^YYFDDKNLYYV**N**STGIDEK^231^ of *Cp*TSP8 (Figs. 4*C*) and ^89^ELKFNGL**N**ITSYENR^103^ of *Cp*TSP11 (Fig. 4*D*), respectively. These minimally processed N-linked glycans are unlike those produced by mammalian, insect, and yeast cells, suggesting that glycoengineered cell lines will be required to produce *C. parvum* antigens with authentic glycosylation profiles.

### Localization of *Cp*TSP proteins in *C. parvum* sporozoites

With the exception of *Cp*TSP8 (*Cp*MIC1) (43), no published localization data exist for the *Cp*TSP family of proteins. While these proteins are all targeted for secretion (Fig. 1*A*) and assumed to be on the cell surface and/or within secretory organelles, confirming their presence on the surface of sporozoites is important if they are to be considered as potential vaccine antigens. To the best of our knowledge, no well-defined antibody tools are available to probe the localization of *Cp*TSP1–12 individually. However, our immunoprecipitation and proteomics experiment (Fig. 3) demonstrated that these proteins are exclusively recognized by 5G12: the C-mannosyl tryptophan–specific mAb (31, 32). Thus, we used this antibody to probe the collective localization of the *Cp*TSP protein family in *C. parvum* sporozoites by immunofluorescence microscopy.

**Figure 5:**
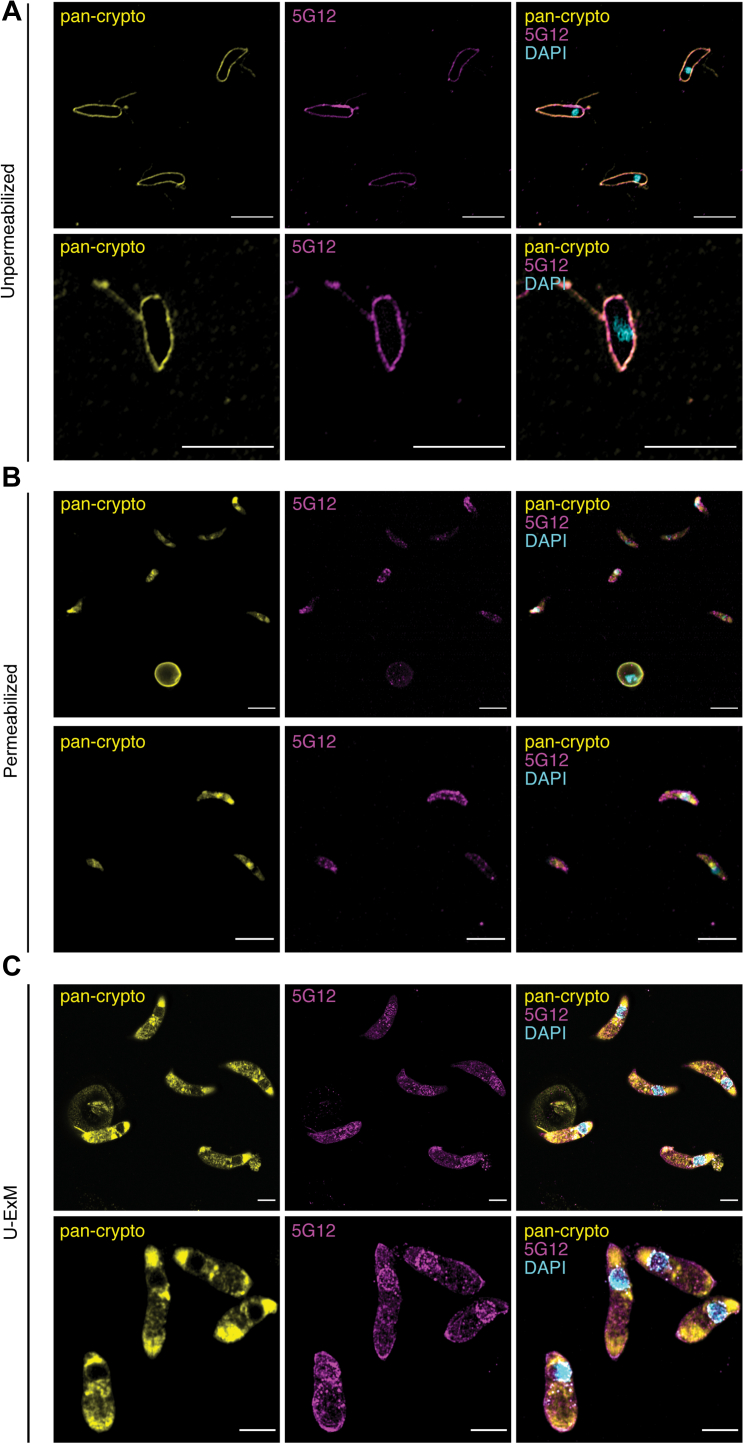
**Immunofluorescence imaging of the *Cp*TSP family within *Cryptosporidium parvum* sporozoites.** Excysted sporozoites were fixed and stained with the “pan-crypto” rabbit serum (*yellow*), the 5G12 monoclonal antibody to tryptophan C-mannosylation (*magenta*); and DAPI (*cyan*). *A*, unpermeabilized sporozoites. *B*, permeabilised sporozoites. *C*, sporozoites subjected to ultrastructural expansion microscopy (U-ExM). Scale bar represents 5 μm. DAPI, 4′,6-diamidino-2-phenylindole.

Initially, 4% paraformaldehyde-fixed and unpermeabilized excysted *C. parvum* sporozoites were probed with the 5G12 mAb, 4′,6-diamidino-2-phenylindole (DAPI) nuclear stain, and a “pan-crypto” serum obtained from a rabbit immunized with *C. parvum* oocyst lysate (Fig. 5*A*). The latter served as a positive control for identifying parasites, immunostaining the intact plasma membrane of all *C. parvum* sporozoites (Fig. 5*A*) as well as residual oocyst wall material (Fig. 5, *B* and *C*). The 5G12 mAb immunostained the intact sporozoite plasma membrane, indicating that one or more of the *Cp*TSP proteins is localized at the sporozoite cell surface.

A similar experiment was performed on fixed and permeabilized sporozoites (Fig. 5*B*). The 5G12 mAb immunostained the cell surface and puncta throughout the cell, which is consistent with the *Cp*TSP family proteins being targeted to secretory structures. To overcome the limited spatial resolution afforded by the small size of *C. parvum* sporozoites (≈2 × 5 μm), we performed ultrastructural expansion microscopy (U-ExM) (44) with the same immunostaining regimen (Fig. 5*C*). For 5G12 immunostaining, we observed surface staining, as well as perinuclear puncta, consistent with localization to endoplasmic reticulum and increased intensity of staining at the apical end of the sporozoite, which is consistent with a micronemal and/or rhoptry localization.

### Localization of *Cp*TSP1 in *C. parvum*

Having established that the *Cp*TSP protein family are collectively localized in the secretory network and on the cell surface of sporozoites, we sought to better understand the distribution of individual protein family members. We were particularly interested in *Cp*TSP1, encoded by *cgd1_3500*, which is otherwise known as ‘thrombospondin related adhesive protein of *Crypsosporidium* 1' (TRAP-C1) (45). Antibodies to *Cp*TSP1 are produced in humans following symptomatic infection with *C. parvum* (46), and this protein is analogous to that of the motility-associated adhesins MIC2 in *T. gondii* (13) and TRAP in *Plasmodium* spp. (14), suggesting that it may have potential as a vaccine antigen candidate.

**Figure 6:**
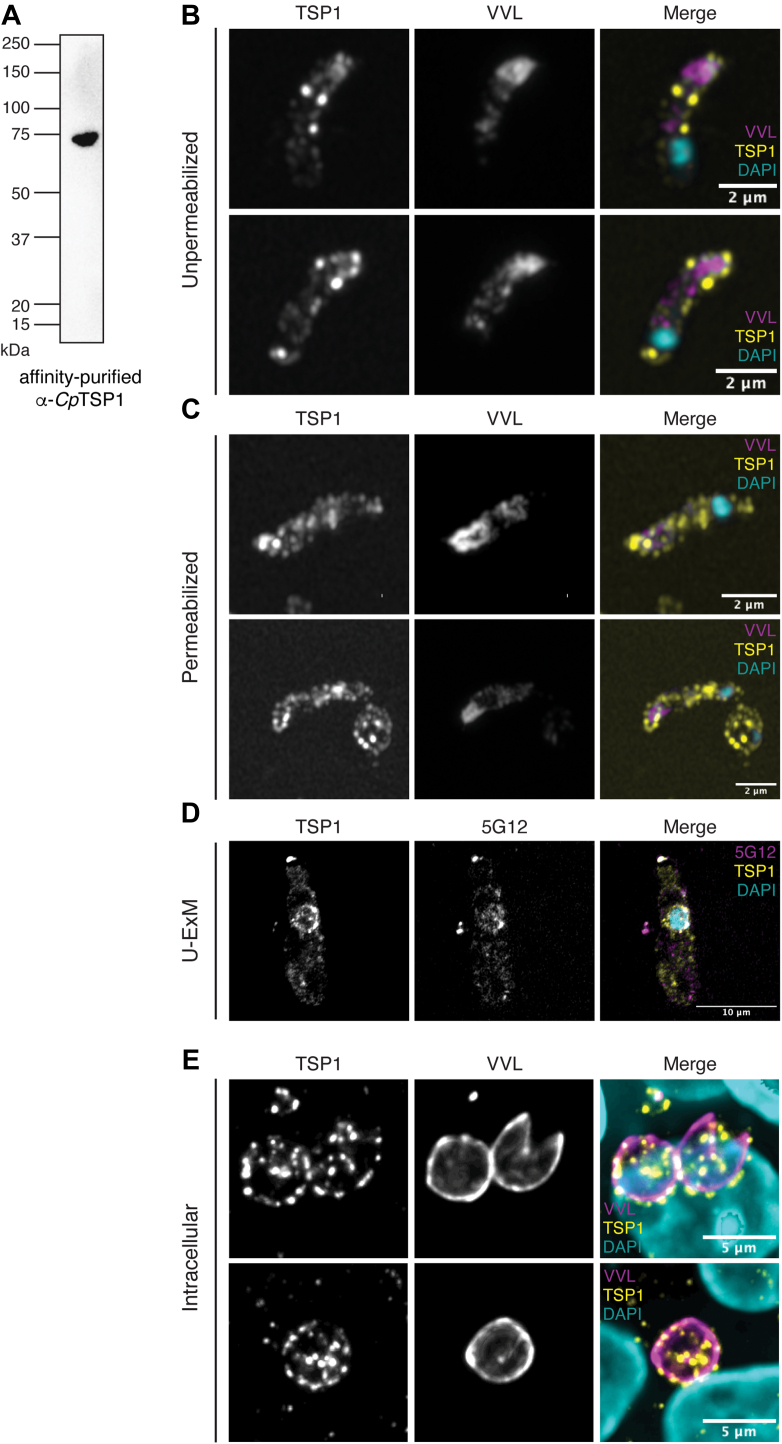
**Localization of *Cp*TSP1 (TRAP-C1) within *Cryptosporidium parvum* sporozoites and meronts.***A*, Western blot analysis of SDS-extracted proteins from *C. parvum* sporozoites using affinity-purified α-*Cp*TSP1 as primary antibody. *B* and *C*, excysted sporozoites were fixed and stained with fluorescein-conjugated *Vicia villosa* lectin (VVL) (*magenta*), α-*Cp*TSP1 antibody (*yellow*), and DAPI (*cyan*). Prior to immunostaining, sporozoites were either (*B*) kept unpermeabilized or (*C*) permeabilized. *D*, excysted sporozoites were subjected to ultrastructural expansion microscopy (U-ExM) and stained with α-*Cp*TSP1 (*yellow*), the 5G12 monoclonal antibody to tryptophan C-mannosylation (*magenta*), and DAPI (*cyan*). *E*, intracellular parasites, 24 h postinfection were fixed, permeabilized, and stained with VVL (*magenta*), anti-*Cp*TSP1 antibody (*yellow*), and DAPI (*cyan*). DAPI, 4′,6-diamidino-2-phenylindole. VVL, *Vicia villosa* lectin.

We recombinantly expressed the third TSR domain of *Cp*TSP1 (*CpTSP1*_372–429_) with an N-terminal Strep tag and C-terminal hexahistidine tag in *E. coli*. After purification, this protein was immobilized on StrepTactin resin and used as bait to affinity-purify antibodies from the polyclonal IgG extracted from the serum of an immunized rabbit. The specificity of this affinity-purified polyclonal antibody for *Cp*TSP1 was assessed by western blot on lysate from *C. parvum* sporozoites: only one band at the expected molecular weight of ≈74 kDa was observed (Figs. 6*A* and S1).

These purified rabbit antibodies were used in a series of imaging experiments to determine where *Cp*TSP1 is localized in sporozoites. This precluded the use of the rabbit “pan-crypto” serum as a control stain for sporozoites: fluorescein-labeled *Vicia villosa* lectin (VVL), which is specific for terminal GalNAc, was used instead (47). Standard fixation of sporozoites and immunostaining using *Cp*TSP1 antibodies without permeabilization demonstrated the presence of *Cp*TSP1 at the apical end of parasites and as puncta across the surface of the parasite (Fig. 6*B*). Following permeabilization, *Cp*TSP1 staining was observed as intracellular puncta throughout the sporozoites (Fig. 6*C*), with a concentration toward the apical end (distal to the nucleus). U-ExM was then employed to resolve clear staining for *Cp*TSP1 around the periphery of the nucleus and at the apical tip: this was largely coincident with 5G12 staining, as expected (Fig. 6*D*).

Finally, to assess if *Cp*TSP1 was relevant to other stages of the life cycle, we stained and imaged intracellular parasites (meronts) 24 h after host cell infection: a time point where asexual stages of the life cycle can be observed. We observed strong punctate staining for *Cp*TSP1 within, and just at the margin of, the parasitophorous vacuole marked by VVL staining (47). Collectively, these imaging data reveal that *Cp*TSP1 is expressed across multiple stages of the *C. parvum* life cycle and is most likely secreted from microneme or rhoptry organelles onto the cell surface.

## Discussion

Amongst the better studied apicomplexan parasites, such as *T. gondii* and *Plasmodium* spp., proteins with TSR domains have repeatedly been identified as critical adhesins for various stages of the parasite life cycle (13, 14, 15, 16, 17, 18) and as promising vaccine antigen candidates (19). The same is likely to be true for the *Cryptosporidium* TSR proteins: a hypothesis that we begin to explore here.

The architecture of the *Cp*TSP protein family suggests that many share a recent common ancestor, and perhaps a common and/or redundant function. For example, *Cp*TSP1,3–6 possess a conserved alternating string of PAN and TSR domains at the N terminus, followed by a variable number of TSR domains. *Cp*TSP1 and *Cp*TSP6 are distinct amongst this group because they possess a C-terminal transmembrane domain, making them type-I integral membrane proteins analogous to gliding motility-associated adhesins like *T. gondii* MIC2 or *Plasmodium* spp. TRAP. MIC2, TRAP, and related apicomplexan adhesins can have complex interactomes: their cytoplasmic domains engage an actomyosin motor complex to drive parasite motility (48), whereas their ectodomains can associate with other parasite proteins to produce large adhesin complexes (49, 50). Identifying other parasite proteins that associate with each member of the *Cp*TSP protein family is an important next step in delineating their function.

Another important step toward understanding the function of these proteins is to determine what host ligands, if any, they recognize. The PAN domains of *Cp*TSP1,3–6 are analogous to those found in *T. gondii* MIC4 (24) and *S. muris* SML-2 (25), where they serve as galactose-binding lectins involved in host cell adhesion. It may be that *Cp*TSP1,3–6 have a similar lectin activity. Lectin-based adhesins may be advantageous for a zoonotic parasite of the intestinal mucosa like *C. parvum*, since this environment is dominated by host mucin glycoproteins adorned with glycan structures that are well conserved in mammals. The generality of such protein–glycan interactions across many host species might also explain the conservation of these proteins across *Cryptosporidium* spp., as well as the presence of galectin-like domains in *Cp*TSP7–9, and a C-type lectin domain in *Cp*TSP11. Determining if these PAN, galectin, and C-type lectin domains bind mucosal glycans, and what structures they recognize, could lead to the identification of function-blocking epitopes.

Beyond the potential importance of host glycans, parasite glycosylation is also likely to play a key role in the function of these proteins and the *C. parvum* life cycle more generally. The O-linked dHexHex glycan detected here on *Cp*TSP1 and *Cp*TSP7 corresponds to the βGlc (1→3)αFuc disaccharide, which is also found in *T. gondii* and *Plasmodium* spp. (34, 37). In *P. falciparum*, O-fucosylation of TSR proteins by POFUT2 is essential for the efficient trafficking of adhesins like TRAP, with disruption of *pofut2* resulting in attenuated transmission to mosquitos, and defects in sporozoite gliding motility, cell traversal, and hepatocyte invasion (34). Similarly, the tryptophan C-mannosylation detected here on *Cp*TSP1–4,7–8,11 has been observed to play important roles in *T. gondii* and *Plasmodium* spp. biology (35, 36, 51). In *T. gondii*, tryptophan C-mannosylation stabilizes the TSR-containing adhesin MIC2 and is thus important for parasite motility (36). In *P. falciparum* and *Plasmodium berghei*, tryptophan C-mannosylation is essential for transmission to the mosquito through its role in stabilization of the TSR-containing adhesins MTRAP and CTRP, which are required for gamete egress and ookinete motility, respectively (35, 51). These findings are commensurate with the general observation that tryptophan C-mannosylation stabilizes proteins with a TSR domain (52, 53). Given the preponderance of tryptophan C-mannosylation sites on the *Cp*TSP protein family, it seems likely that this protein modification will be important to several aspects of the parasite’s life cycle, and that the dpy-19 enzyme that installs this protein modification may have potential as a novel drug target. Indeed, apicomplexan dpy-19 enzymes are among the most divergent from mammalian homologs, and structural data for this enzyme family are now available (54), suggesting that it may be possible to develop selective inhibitors of the *C. parvum* dpy-19 homolog. Furthermore, the molecular genetic techniques needed to validate this potential drug target are now available (55).

The case for TSR domain–containing proteins in apicomplexans as vaccine antigens remains strong, with GSK’s RTS,S/AS01 still being the only approved *P. falciparum* vaccine (19). While RTS,S offers only modest protection, reformulation of its protein antigen, which is expressed in yeast, with an alternative adjuvant recently delivered greatly improved protection in clinical trials (56). We have demonstrated that analogous TSR-containing proteins are well conserved in *C. parvum*, expressed in sporozoites and meronts, and localized on the surface and in the secretory pathway, making them worthy candidates for further exploration as vaccine antigens. Our glycoproteomic data sets have substantially built on earlier experiments (39), confirming that minimally processed Hex_5–6_HexNAc_2_ structures dominate in *C. parvum*. Producing antigen with a similar glycosylation profile *in vivo*, for example with mRNA or adeno-associated virus vectors, is not possible (57). To recapitulate native N-linked glycan profiles, protein antigen will need to be heterologously produced in a glycoengineered cell line, such as the *Pichia pastoris* (*Komagataella pastoris*) (58). While further engineering will be required to introduce the relevant C-mannosylation (31) and O-fucosylation (59) pathways, doing so would afford a platform for the low-cost production of high-quality *Cryptosporidium* antigens with native glycosylation profiles.

## Conclusion

This work has provided new insights into the architecture, conservation, relative abundance, glycosylation, and localization of the *Cp*TSP family of proteins in *C. parvum* sporozoites. These proteins, which are orthologous to other important apicomplexan adhesins, have significant potential as vaccine antigen candidates. They are both well conserved in *C. parvum* populations and highly similar to orthologs in *C. hominis*, another important human pathogen. Some of these proteins, particularly *Cp*TSP1, are expressed at high levels in sporozoites, present on the cell surface, and localized in patterns reminiscent of other apicomplexan motility–associated adhesins. Two glycopeptide enrichment strategies coupled with protein mass spectrometry enabled a characterization of the native post-translational modifications on the *Cp*TSP protein family. This revealed that *C. parvum* performs tryptophan C-mannosylation and O-fucosylation of its TSR domains, akin to metazoans and other apicomplexans, and confirmed that the parasite’s N-glycans are of a minimally processed (Hex_5–6_HexNAc_2_) nature, which differs to those commonly produced by mammalian, insect, and yeast cell lines. This work sets the stage for further exploration of the biology of this protein family and their potential use as vaccine antigens.

## Experimental procedures

### Protein domain boundary assignment using AlphaFold2

AlphaFold2 (20) was used to build models of *Cp*TSP1–12 (UniProt IDs: Q5CSA5, Q5CRC0, Q5CSA4, Q5CQ00, Q5CXF3, Q5CX66, Q5CQ18, Q5CXK1, Q5CXK0, Q5CTG7, Q5CXC2, and Q5CPW4, respectively) and the boundaries of globular domains assigned by manual inspection (Fig. 1*A* and Table S1). Hitherto unannotated domains were classified by performing structural homology searches using DALI (23) and a Z-score cutoff >2.

### Molecular evolution and conservation

Raw sequencing reads of 32 *C. parvum* genomes (60, 61, 62) were retrieved from the European Nucleotide Archive repository; filtered low-quality bases and trimmed adapters using Trimmomatic v.0.36 (63) and mapped to the *C. parvum* IOWA-ATCC reference genome (64) using BWA-MEM v.0.7 (65). SNPs were identified based on the variant calling protocol described in the study by Tichkule *et al.* (66). Population genetic indices (nucleotide diversity, number of segregating sites, and Tajima D values) were calculated by using PopGenome R package (67).

### Protein extraction

Approximately 2 × 10^9^ *C. parvum* oocysts (Bunch Grass Farm) were bleached for 10 min on ice with 1.75% sodium hypochlorite, followed by excystation in 0.8% sodium deoxytaurocholate (Sigma) for 10 min at 37 °C and then PBS for 1 h at 37 °C, 5% CO_2_. Excysted parasites were washed with PBS and resuspended in lysis buffer (50 mM Tris [pH 7.50], 150 mM NaCl, 0.1% SDS, 0.5% sodium deoxycholate, 1% Triton X-100 supplemented with 1× protease inhibitor cocktail [Roche], and 1× benzonase [Merck]). The lysate was incubated on ice for 30 min with vortexing every 5 min. The protein concentration of the crude lysate was quantitated using a bicinchoninic acid assay. An aliquot of the lysate containing 10 mg of protein was made up to 200 μl with MilliQ H_2_O in a 1.5 ml microcentrifuge tube, then 800 μl of acetone was added, and the mixture was stored for 16 h at −20 °C. The precipitate was pelleted by centrifugation (6000*g*, 15 min, 4 °C), and the supernatant was discarded. The pellet was resuspended in 200 μl H_2_O, transferred to a fresh 1.5 ml microcentrifuge tube, 800 μl of acetone was added, and the mixture was kept for 4 h at −20 °C. The precipitate was pelleted by centrifugation (6000*g*, 15 min, 4 °C), the supernatant was discarded, and the pellet air-dried for 1 h at 22 °C.

### Trypsin digestion

The protein pellet was resuspended in 100 μl denaturation buffer (20 mM NH_4_HCO_3_, 6 M urea, and 2 M thiourea) with vortexing and the protein concentration was redetermined by bicinchoninic acid assay. DTT (1 μl, 1 M) was added, and the sample was nutated for 60 min at 22 °C to complete peptide dissolution. 2-Chloroacetamide (50 μl, 100 mM) was added, and the sample was nutated with the exclusion of light for 60 min at 22 °C. The alkylation reaction was quenched with more DTT (4 μl, 1 M) and nutated for 10 min at 22 °C. The sample was diluted with 465 μl of 100 mM NH_4_HCO_3_ before the addition of 20 μg trypsin (Promega) and incubation for 16 h at 25 °C and 500 rpm. The sample was acidified by the addition of 20 μl HCO_2_H, centrifuged (10,000*g*, 10 min, 22 °C), and the supernatant was applied to a 50 mg tC18 Sep-Pak column (Waters) conditioned in buffer A (0.1% TFA, 2% MeCN, and 97.9% H_2_O). The column was washed with buffer A (3 × 800 μl), eluted with 800 μl buffer B (0.1% TFA, 80% MeCN, and 19.9% H_2_O), and the eluate was dried on a SpeedVac system (Thermo Fisher Scientific) and then stored at −20 °C until further use.

### FAIMS-based proteomic analysis

To enable deep proteomic analysis, FAIMS-based fractionation was undertaken. About 20 μg of *C. parvum* proteome samples were resuspended in buffer A∗ (2% acetonitrile and 0.1% TFA), and 2 μg of peptide was used for each FAIMS column volume (CV). Peptide samples were separated using a two-column chromatography set up composed of a PepMap100 C18 20 mm × 75 μm trap and a PepMap C18 500 mm × 75 μm analytical column (Thermo Fisher Scientific). Samples were concentrated onto the trap column at 5 μl.min^−1^ for 5 min with buffer A (0.1% formic acid and 2% dimethyl sulfoxide [DMSO]) and then infused into an Orbitrap Fusion Lumos Tribrid Mass Spectrometer (Thermo Fisher Scientific) equipped with an FAIMS Pro interface at 300 nl.min^−1^ *via* the analytical column using a Dionex Ultimate 3000 UPLC (Thermo Fisher Scientific). About 125 min analytical runs were undertaken by altering the buffer composition from 2% buffer B (0.1% formic acid, 77.9% acetonitrile, and 2% DMSO) to 23% B over 95 min, then from 23% B to 40% B over 10 min, and then from 40% B to 80% B over 7 min. The composition was held at 80% B for 3 min and then dropped to 2% B over 1 min before being held at 2% B for another 9 min. The Lumos Mass Spectrometer was operated in a static FAIMS data-dependent mode automatically switching between the acquisition of a single Orbitrap MS scan (120 k resolution) every 3 s and HCD MS2 events (FTMS, 15 k resolution, maximum fill time 80 ms, normalized collision energy (NCE) of 30, and automatic gain control [AGC] of 250%). A total of seven FAIMS CV were acquired: −20, −30, −40, −50, −60, −70, −80, and −90. Oxonium ions (204.0867; 138.0545 and 366.1396 *m/z*) product-dependent MS/MS analysis (68) was used to trigger three additional scans of potential glycopeptides; an Orbitrap EThcD scan (NCE = 15%, maximal injection time = 250 ms, AGC = 2 × 10^5^ with a resolution of 30 k and using the extended mass range setting to improve the detection of high mass glycopeptide fragment ions) (69); a ion trap collision-induced dissociation scan (NCE = 35%, maximal injection time = 40 ms, and AGC 5 × 10^4^) and a stepped collision energy HCD scan (using NCE 35% with 8% stepping, maximal injection time = 150 ms, AGC 2 × 10^5^ with a resolution of 30 k).

### Immunoprecipitation of C-mannosylated peptides

5G12 and an isotype control IgG (100 μg) were separately incubated with protein G agarose beads (500 μl of a 50% suspension) in immunoprecipitation (IP) buffer (50 mM Mops, pH 7.2, 50 mM NaCl, and 10 mM Na_3_PO_4_) for 16 h at 4 °C. The agarose beads were collected in a spin cup (Pierce) by centrifugation (500*g*, 5 min, 4 °C) and washed three times with 500 μl IP buffer. The purified tryptic peptides were resuspended in 5 ml IP buffer, and 500 μl samples were added to 10 microcentrifuge tubes. Five tubes were treated with 100 μl of the 5G12-coupled agarose beads, and the other five tubes were treated with the 100 μl of the isotype control–coupled beads, then the samples were nutated for 4 h at 22 °C. The beads from each sample were collected in a spin cup (Pierce) by centrifugation (500*g*, 5 min, 4 °C) and washed five times with 500 μl IP buffer. Peptides were eluted from the beads using two consecutive treatments with 200 μl 0.2% TFA in MilliQ H_2_O. Peptides from each of the five treatment and control samples were captured from solution using C_18_ stage tips, eluted using 0.1% HCO_2_H/MeCN 1:4, dried and stored at −20 °C prior to analysis by LC–MS.

### LC–MS analysis of immunoprecipitated peptides

Enriched peptide samples were resuspended in buffer A∗ (2% acetonitrile and 0.1% TFA) and separated using a two-column chromatography setup comprised of a PepMap100 C18 20 mm × 75 μm trap column and a PepMap C18 500 mm × 75 μm analytical column (Thermo Fisher). Samples were concentrated onto the trap column at 5 μl.min^−1^ for 5 min with buffer A (0.1% formic acid and 2% DMSO) and then infused into an Orbitrap 480 Mass Spectrometer (Thermo Fisher) at 300 nl.min^−1^ *via* the analytical column using a Dionex Ultimate 3000 UPLC (Thermo Fisher). Analytical runs 125 min long were undertaken by altering the buffer composition from 2% buffer B (0.1% HCO_2_H, 77.9% MeCN, 2% DMSO, and 20% H_2_O) to 23% B over 95 min, then from 23% B to 40% B over 10 min, and then from 40% B to 80% B over 5 min. The composition was held at 80% B for 5 min and then dropped to 2% B over 1 min before being held at 2% B for another 9 min. The mass spectrometer was operated in a data-dependent mode automatically switching between the acquisition of a single Orbitrap MS scan (maximum injection time = 25 ms, AGC = 3 × 10^6^, 120 k resolution) and MS/MS events for up to 3 s (using stepped NCE = 27; 32; 36%, maximal injection time = 65 ms, AGC = 400%, and 30 k resolution). To further improve the assignments of modified glycopeptides, samples were re-run and MS/MS setting altered to allow a maximal injection time of 120 ms, an AGC of 600%, and a 45 k resolution.

### Analysis of immunoprecipitated peptides and FAIMS-fractionated proteome MS data

Glycopeptides enriched by 5G12 immunoprecipitation and FAIM fractionated proteome samples were analyzed using MaxQuant (v1.6.3.4) (70). Searches were performed against two *C. parvum* IOWA strain databases (UniProt accession: UP000006726 and CryptoDB, version 48) with carbamidomethylation of cysteine set as a fixed modification for 5G12 immunoprecipitation. The variable modifications, oxidation of methionine (M), Hex (W), and dHexHex (S/T), were used, whereas for FAIMS fractionated samples, oxidation of methionine (M) and acetylation of the N termini was used. Searches were performed with trypsin cleavage specificity allowing two missed cleavage events. The precursor mass tolerance was set to 20 ppm for the first search and 10 ppm for the main search, with a maximum false discovery rate of 1.0% set for protein and peptide identifications. To enable the assessment of relative protein abundance, the intensity-based absolute quantification option was enabled for the analysis of FAIMS fractionated samples. For 5G12 immunoprecipitations, the “match between run” (71) setting was enabled to improve the detection of peptides between samples. The output protein group was processed within the Perseus (v1.4.0.6) (72) analysis environment to remove reverse matches and common protein contaminates prior to quantitative analysis using the peptide ion intensities. Missing values were imputed based on the observed total peptide intensities with a range of 0.3σ and a downshift of 2.0σ. Samples were grouped based on the antibody used for the enrichment (5G12 or isotype control). The Student's *t* test was used to assign *p* values, and multiple hypothesis correction was undertaken using a Benjamini–Hochberg correction. To aid in the analysis of the MS/MS of glycopeptides of interest, the Interactive Peptide Spectral Annotator was used (73).

### ZIC-HILIC–MS/MS analysis of trypsin-digest lysate

ZIC-HILIC enrichment was performed as previously described with minor modifications (74). Briefly, a ZIC-HILIC Stage-tip (75) was created by packing 0.5 cm of 10 μm ZIC-HILIC resin (Millipore) into p200 tips containing a frit of C8 Empore (Sigma) material. Prior to use, the column was washed with ultrapure water, followed by 95% acetonitrile and then equilibrated with 80% acetonitrile and 1% TFA. The digested proteome sample was resuspended in 80% acetonitrile and 1% TFA. The whole proteome digest was adjusted to a concentration of 2 μg/μl (a total of 200 μg of peptide used for each enrichment) and then loaded onto equilibrated ZIC-HILIC columns. ZIC-HILIC columns were washed with 20 bed volumes of 80% acetonitrile and 1% TFA to remove nonglycosylated peptides and bound peptides eluted with 10 bed volumes of ultrapure water. Eluted peptides were dried by vacuum centrifugation and stored at −20 °C.

The ZIC-HILIC–enriched sample was resuspended in buffer A∗ (2% acetonitrile and 0.1% TFA) and separated using a two-column chromatography set up composed of a PepMap100 C18 20 mm × 75 μm trap and a PepMap C18 500 mm × 75 μm analytical column (Thermo Fisher Scientific). Samples were concentrated onto the trap column at 5 μl/min for 5 min with buffer A (0.1% formic acid and 2% DMSO) and then infused into an Orbitrap Fusion Lumos Tribrid Mass Spectrometer (Thermo Fisher Scientific) equipped with an FAIMS Pro interface at 300 nl/min *via* the analytical column using a Dionex Ultimate 3000 UPLC (Thermo Fisher Scientific). About 185 min analytical runs were undertaken by altering the buffer composition from 2% buffer B to 23% B over 155 min, then from 28% B to 45% B over 12 min, and then from 45% B to 80% B over 5 min. The composition was held at 80% B for 3 min and then dropped to 2% B over 1 min before being held at 2% B for another 9 min. The Lumos Mass Spectrometer was operated in a stepped FAIMS data-dependent mode automatically switching between the acquisition of a single Orbitrap MS scan (120 k resolution) every 2 s and HCD MS2 events (FTMS, 15 k resolution, maximum fill time 80 ms, NCE 30, and AGC of 250%) at three different FAIMS CVs −25, −45, and −65 as previously described (76). Oxonium ion (204.0867, 138.0545, and 366.1396 *m/z*) product-dependent MS/MS analysis (68) was used to trigger three additional scans of potential glycopeptides; an Orbitrap EThcD scan (NCE = 15%, maximal injection time = 250 ms, AGC = 2 × 10^5^ with a resolution of 30 k, and using the extended mass range setting to improve the detection of high mass glycopeptide fragment ions (69)); a ion trap collision-induced dissociation scan (NCE = 35%, maximal injection time = 40 ms, and AGC = 5 × 10^4^) and a stepped collision energy HCD scan (using NCE 35% with 8% stepping, maximal injection time = 150 ms, and AGC = 2 × 10^5^ with a resolution of 30 k).

### Analysis of MS data for ZIC-HILIC–enriched glycopeptides

ZIC-HILIC–enriched glycopeptides were identified using glycosylation enabled MSFragger ((77, 78) version 14.0) searching against the *C. parvum* (strain Iowa II) database (UniProt: UP000006726, 3805 proteins downloaded October 11, 2020). The resulting data were visualized using ggplot2 within R by tallying the observed delta masses of identified glycopeptides. To aid in the analysis of the MS/MS of glycopeptides of interest, the Interactive Peptide Spectral Annotator was used (73).

### Generation of “pan crypto” rabbit serum

Rabbits were handled in accordance with the guidelines of the National Health and Medical Research Committee and the PHS Policy on Humane Care and Use of Laboratory Animals. Details of our procedures were approved by the WEHI Animal Welfare Committee, approval number 2020.019. Rabbits were immunized with 200 μg of *C. parvum* sporozoite lysate and Freund’s complete adjuvant. They subsequently received two boosters of 200 μg *C parvum* sporozoite lysate with Freund’s incomplete adjuvant.

### Ultrastructural expansion microscopy

Purified *C. parvum* oocysts were obtained as previously described (79) and sedimented onto coverslips coated with polylysine (catalog no.: A3890401; Thermo) through centrifugation at 250*g* for 3 min at room temperature. Parasites were fixed with methanol at −20 °C for 7 min and expanded using U-ExM as previously published (44). Briefly, coverslips were incubated for 5 h in 0.7% acrylamide (AA)/1% FA mix at 37 °C and transferred to a wet chamber with monomer solution (19% sodium acrylate; 10% AA; 0.1% Bis-AA in PBS 10×) supplemented with 0.5% APS and 0.5% *N*, *N*, *N*', *N*' - tetramethylethylenediamine for 1 h at 37 °C. Next, coverslips with gels were incubated in denaturation buffer (200 mM SDS, 200 mM NaCl, and 50 mM Tris in ddH_2_O, pH 9) for 15 min at room temperature with gentle agitation. Forceps were used to remove the gels from the coverslips and transferred to tubes with fresh denaturation buffer at 95 °C for 90 min (80). Gels were washed with water 2× for 30 min and left to expand overnight. Prior to immunostaining, gels were washed twice for 15 min with PBS and then incubated for 3 h at 37 °C with primary antibodies 5G12, *Cp*TSP1, or Pan-Crypto. DAPI was incubated together with the secondaries (1:500 dilution). Gels were washed three times for 10 min in PBS–Tween 0.1% prior to incubation with secondary antibodies (antimouse Alexa 488, antimouse Alexa 594, antimouse Alexa 647, anti-rabbit Alexa 488, anti-rabbit Alexa 594, and anti-rabbit Alexa 647) during 3 h at 37 °C, followed by three washes of 10 min in PBS–Tween. A second round of expansion was performed overnight in water before imaging. Imaging was performed on a Zeiss LSM 880 confocal microscope using Fast Airyscan with a 63× 1.4 numerical aperture oil objective. Images were edited using ImageJ software.

### Immunofluorescence microscopy

Parasites were prepared as described previously and fixed with 4% (v/v) formaldehyde in PBS for 10 min. Permeabilization was performed with 0.1% Triton X-100 in PBS for 10 min and blocking with 3% bovine serum albumin (BSA) in PBS for 10 min. Cells were incubated with primary antibodies 5G12, *Cp*TSP1, or Pan-Crypto diluted in 3% BSA in PBS for 1 h followed by 3× 10 min washes with PBS. Secondary antibodies, VVL (fluorescein conjugated; Vector Laboratories, FL-1231-2) and DAPI, were diluted in 3% BSA in PBS, incubated for 1 h at room temperature, washed 3× for 10 min with PBS, and mounted in Vectashield (Vector Laboratories). For unpermeabilized samples, incubation with 0.1% Triton X-100 was not performed. Images were taken on an Opera Phenix high content imaging platform (PerkinElmer) using 63× objective. Data were processed in Fiji to yield maximum projection images.

### Production of recombinant *Cp*TSP1_372–429_

A dsDNA oligonucleotide encoding residues 371 to 429 of *Cp*TSP1 (UniProt: Q5CSA5) that had been codon-harmonized for expression in *E. coli* was synthesized (IDT) and cloned into the pET29b(+) (Novagen) expression vector using the NdeI/NotI restriction sites (Table S3). The resulting plasmid, after verification by Sanger sequencing, was transformed into chemically competent “SHuffle T7” *E. coli* cells (NEB) and transformants selected on LB-agar (50 μg ml^−1^ Kan) by incubation at 37  °C for 16 h. A single colony was used to inoculate 10 ml of LB media containing 50 μg ml^−1^ Kan, and the culture was incubated at 37  °C for 16 h. This starter culture was used to inoculate 600 ml of S-broth (35 g tryptone, 20 g yeast extract, 5 g NaCl, pH 7.4) containing 50 μg ml^−1^ Kan, which was incubated with shaking (250 rpm) at 37  °C until it reached an absorbance at 600 nm of 0.7. After cooling to room temperature, isopropyl thiogalactoside added to a final concentration of 0.4 M, and incubation with shaking (200 rpm) continued at 18  °C for 16 h. Cells were harvested by centrifugation at 8000*g* for 20 min at 4  °C and then resuspended in 40 ml binding buffer (50 mM NaP_i_, 300 mM NaCl, 5 mM imidazole, pH 7.5) containing protease inhibitor (Roche cOmplete EDTA-free protease inhibitor mixture) and lysozyme (0.1 mg ml^−1^) by nutating at 4  °C for 30 min. Benzonase (1 μl, 250 U) was added to the mixture and then lysis was effected by sonication (10 × [15 s on/45 s off] at 45% amplitude). The lysate was centrifuged at 18,000*g* for 20 min at 4 °C, and the supernatant was collected. The supernatants were filtered (0.45 μm) and loaded onto a 1 ml HisTrap column (GE). The column was washed with 3 × 10 ml of binding buffer, and then the protein was eluted using elution buffer (50 mM NaP_i_, 300 mM NaCl, 400 mM imidazole, pH 7.5). Fractions containing product, as judged by SDS-PAGE, were further purified by size-exclusion chromatography on a Superdex 75 Increase 10/300 GL column (GE) using 50 mM NaP_i_, 150 mM NaCl, pH 7.5.

### Affinity purification of *Cp*TSP1-reactive rabbit IgG

Protein A agarose resin (1 ml of 50% slurry; Sigma–Aldrich) was loaded into a gravity flow column (Bio-Rad) and equilibrated with five CVs of TBS buffer (25 mM Tris, 150 mM NaCl, pH 7.5). “Pan-crypto” rabbit serum (10 ml) was passed through the column, and the column was washed with 10 CVs of TBS buffer. The bound IgG was eluted using 10 CVs of 100 mM glycine (pH 3.0) buffer into microcentrifuge tubes containing 1 CV of 2 M Tris, 1 M NaCl, pH 8.0 buffer. Recombinant *Cp*TSP1_372–429_ protein in TBS buffer (25 mM Tris, 150 mM NaCl, pH 7.5) was passed through a 1 ml Strep-Tactin XL superflow column (IBA-Lifesciences) to saturate the resin with this “bait” protein. The purified IgGs were then passed through this column, and the nonreactive IgGs were removed with 50 CVs of washing buffer (100 mM Tris, 150 mM NaCl, 1 mM EDTA, pH 8.0). The bound *Cp*TSP1-reactive antibodies were subsequently eluted using 10 CVs of 100 mM glycine, pH 10 buffer into microcentrifuge tubes containing 1 CV of 1 M Tris, pH 7.5 buffer. The sample was concentrated to 1 mg ml^−1^ and flash frozen as 10 μl aliquots until further use.

## Data availability

Proteomics data have been deposited to the ProteomeXchange Consortium *via* the PRIDE partner repository with the dataset identifiers PXD023935 (FAIMS data), PXD023936 (ZIC-HILIC-enriched samples) and PXD023934 (5G12-enriched samples).

## Supporting information

This article contains supporting information.

## Conflict of interest

The authors declare that they have no conflicts of interest with the contents of this article.
