# Supplementary material for: Conservation, abundance, glycosylation profile, and localization of the TSP protein family in Cryptosporidium parvum
Source: J Biol Chem. 2023 Feb 10;299(3):103006. doi: 10.1016/j.jbc.2023.103006 (PMC10034466; doi:10.1016/j.jbc.2023.103006)
Supplement: Supplementary Information [file mmc1.docx]

**SUPPLEMENTARY INFORMATION**

**Conservation, abundance, glycosylation profile, and localization of the TSP protein family in *Cryptosporidium parvum***

Alan John^1,2†^, Stefanie M. Bader^1,2†^, Niccolay Madiedo Soler^1,2^, Kharizta Wiradiputri^1,2^, Swapnil Tichkule^1,2^, Sean T. Smyth^1,2^, Stuart A. Ralph^3^, Aaron R. Jex^1,2^, Nichollas E. Scott^4*^, Christopher J. Tonkin^1,2*^, Ethan D. Goddard-Borger^1,2*^

^1^ The Walter and Eliza Hall Institute of Medical Research, Parkville, Victoria 3052, Australia.

^2^ Department of Medical Biology, University of Melbourne, Parkville, Victoria 3010, Australia.
^3^ Department of Biochemistry and Pharmacology, Bio21 Molecular Science and Biotechnology Institute, University of Melbourne, Parkville, Victoria 3010, Australia.

^4^ Department of Microbiology and Immunology, University of Melbourne at the Peter Doherty Institute for Infection and Immunity, Parkville, Victoria 3010, Australia.

† these authors contributed equally

* to whom correspondence should be addressed

**Table S1.** The domain boundaries assigned to each *Cp*TSP protein family member with the aid of Alphafold2 models.

| **gene** | **protein** | **UniProt accession** | **predicted domains** | | **No. of TSRs** |
| --- | --- | --- | --- | --- | --- |
| cgd1_3500 | *Cp*TSP1 | [Q5CSA5](https://www.uniprot.org/uniprot/Q5CSA5) | 1–18 | signal peptide | 6 |
|  | (TRAP-C1) |  | 66–151 | PAN domain |  |
|  |  |  | 155–206 | TSR domain |  |
|  |  |  | 210–308 | PAN domain |  |
|  |  |  | 313–371 | TSR domain |  |
|  |  |  | 374–427 | TSR domain |  |
|  |  |  | 432–478 | TSR domain |  |
|  |  |  | 485–556 | TSR domain |  |
|  |  |  | 560–609 | TSR domain |  |
|  |  |  | 621–644 | transmembrane domain |  |
| cgd5_3420 | *Cp*TSP2 | [Q5CRC0](https://www.uniprot.org/uniprot/Q5CRC0) | 1–23 | signal peptide | 14 |
|  | (TRAP-C2/3) |  | 38-94 | LNR domain |  |
|  |  |  | 97-130 | LNR domain |  |
|  |  |  | 135-189 | TSR domain |  |
|  |  |  | 193-245 | TSR domain |  |
|  |  |  | 249-307 | TSR domain |  |
|  |  |  | 311-367 | TSR domain |  |
|  |  |  | 381-442 | TSR domain |  |
|  |  |  | 447-493 | TSR domain |  |
|  |  |  | 508-574 | TSR domain |  |
|  |  |  | 582-645 | TSR domain |  |
|  |  |  | 649-741 | TSR domain |  |
|  |  |  | 745-795 | TSR domain |  |
|  |  |  | 799-855 | TSR domain |  |
|  |  |  | 859-910 | TSR domain |  |
|  |  |  | 914-964 | TSR domain |  |
|  |  |  | 970-1021 | TSR domain |  |
|  |  |  | 1026-1083 | Shkt |  |
|  |  |  | 1086-1116 | LNR domain |  |
|  |  |  | 1301-1401 | streptavidin-like domain |  |
|  |  |  | 1480-1529 | Shkt |  |
|  |  |  | 1532-1562 | LNR domain |  |
|  |  |  | 1632-1684 | Shkt |  |
|  |  |  | 1687-1717 | LNR domain |  |
|  |  |  | 1763-1821 | LNR domain |  |
|  |  |  | 1861-1910 | Shkt |  |
|  |  |  | 1913-1943 | LNR domain |  |
|  |  |  | 2011-2067 | LNR domain |  |
|  |  |  | 2092-2155 | LNR domain |  |
|  |  |  | 2159-2219 | Shkt domain |  |
|  |  |  | 2396-2453 | Shkt |  |
|  |  |  | 2801-2835 | LNR domain |  |
|  |  |  | 3096-3126 | LNR domain |  |
|  |  |  | 3445-3486 | SCR domain |  |
|  |  |  | 3487-3556 | SCR domain |  |
| cgd1_3510 | *Cp*TSP3 | [Q5CSA4](https://www.uniprot.org/uniprot/Q5CSA4) | 1–22 | signal peptide | 4 |
|  |  |  | 23–107 | PAN domain |  |
|  |  |  | 111–158 | TSR domain |  |
|  |  |  | 200–289 | PAN domain |  |
|  |  |  | 293–348 | TSR domain |  |
|  |  |  | 353–416 | TSR domain |  |
|  |  |  | 421–460 | TSR domain |  |
| cgd8_150 | *Cp*TSP4 | [Q5CQ00](https://www.uniprot.org/uniprot/Q5CQ00) | 1–25 | signal peptide | 2 |
|  |  |  | 70–155 | PAN domain |  |
|  |  |  | 159–210 | TSR domain |  |
|  |  |  | 214–299 | PAN domain |  |
|  |  |  | 303–355 | TSR domain |  |
| cgd6_1300 | *Cp*TSP5 | [Q5CXF3](https://www.uniprot.org/uniprot/Q5CXF3) | 1–28 | signal peptide | 2 |
|  |  |  | 37–123 | PAN domain |  |
|  |  |  | 127–181 | TSR domain |  |
|  |  |  | 185–291 | PAN domain |  |
|  |  |  | 295–343 | TSR domain |  |
| cgd6_2310 | *Cp*TSP6 | [Q5CX66](https://www.uniprot.org/uniprot/Q5CX66) | 1–18 | signal peptide | 2 |
|  |  |  | 52–99 | TSR domain |  |
|  |  |  | 112–190 | PAN domain |  |
|  |  |  | 193–250 | TSR domain |  |
|  |  |  | 279-300 | transmembrane domain |  |
| cgd5_4470 | *Cp*TSP7 | [Q5CQ18](https://www.uniprot.org/uniprot/Q5CQ18) | 12–32 | transmembrane domain | 3 |
|  |  |  | 235–360 | galectin-like domain |  |
|  |  |  | 361–413 | TSR domain |  |
|  |  |  | 416–466 | TSR domain |  |
|  |  |  | 470–521 | TSR domain |  |
|  |  |  | 525–557 | EGF-like domain |  |
|  |  |  | 563–582 | transmembrane domain |  |
| cgd6_780 | *Cp*TSP8 | [Q5CXK1](https://www.uniprot.org/uniprot/Q5CXK1) | 14–31 | transmembrane domain | 3 |
|  | (*Cp*MIC1) |  | 32–166 | galectin-like domain |  |
|  |  |  | 391–443 | TSR domain |  |
|  |  |  | 446–494 | TSR domain |  |
|  |  |  | 498–550 | TSR domain |  |
|  |  |  | 554–589 | EGF-like domain |  |
|  |  |  | 597–620 | transmembrane domain |  |
| cgd6_800 | *Cp*TSP9 | [Q5CXK0](https://www.uniprot.org/uniprot/Q5CXK0) | 1–26 | signal peptide | 3 |
|  |  |  | 51–189 | galectin-like domain |  |
|  |  |  | 190–242 | TSR domain |  |
|  |  |  | 245–296 | TSR domain |  |
|  |  |  | 300–354 | TSR domain |  |
|  |  |  | 358–391 | EGF-like domain |  |
|  |  |  | 398–416 | transmembrane domain |  |
| cgd2_3080 | *Cp*TSP10 | [Q5CTG7](https://www.uniprot.org/uniprot/Q5CTG7) | 1–27 | signal peptide | 4 |
|  |  |  | 44–102 | TSR domain |  |
|  |  |  | 106–157 | TSR domain |  |
|  |  |  | 160–240 | Kringle domain |  |
|  |  |  | 247–300 | TSR domain |  |
|  |  |  | 304–355 | TSR domain |  |
|  |  |  | 364–385 | transmembrane domain |  |
| cgd6_1660 | *Cp*TSP11 | [Q5CXC2](https://www.uniprot.org/uniprot/Q5CXC2) | 1–32 | signal peptide | 4 |
|  |  |  | 403–468 | TSR domain |  |
|  |  |  | 469–529 | TSR domain |  |
|  |  |  | 532–576 | TSR domain |  |
|  |  |  | 616–714 | C-type lectin domain |  |
|  |  |  | 717-776 | TSR domain |  |
|  |  |  | 786-894 | STAS domain |  |
| cgd8_540 | *Cp*TSP12 | [Q5CPW4](https://www.uniprot.org/uniprot/Q5CPW4) | 1–19 | signal peptide | 4 |
|  |  |  | 128-278 | immunoglobulin-like domain |  |
|  |  |  | 283-332 | TSR domain |  |
|  |  |  | 336-411 | TSR domain |  |
|  |  |  | 418-492 | TSR domain |  |
|  |  |  | 495-741 | immunoglobulin-like domain |  |
|  |  |  | 745-804 | TSR domain |  |

**Table S2.** Population genetic indices of TSP proteins in *C. parvum* across 32 isolates. Segregating sites = number of positions which show difference between gene sequences (of individual samples) in sequence alignment. Nucleotide diversity = measure of genetic variation within population. Tajima’s D = test to detect genes that are not evolving neutrally (Tajima’s D values deviating from 0). Tajima’s D < 0 represents genes under purifying selection (removing non-beneficial mutations from the population). Tajima’s D > 0 represents genes under balancing selection (maintain the beneficial mutation within the population).

| gene (protein) | length | segregating sites | nucleotide diversity | Tajima's D |
| --- | --- | --- | --- | --- |
| cgd8_540 (TSP12) | 2478 | 2 | 0.00022 | 0.17158 |
| cgd6_2310 (TSP6) | 1077 | 1 | 0.00012 | -0.76373 |
| cgd6_1660 (TSP11) | 3381 | 1 | 0.00004 | -0.76373 |
| cgd1_3500 (TSP1) | 2064 | 3 | 0.0003 | -0.44585 |
| cgd1_3510 (TSP3) | 1524 | 3 | 0.00043 | -0.31011 |
| cgd6_1300 (TSP5) | 1065 | 0 | 0 | N/A |
| cgd8_150  (TSP4) | 1467 | 0 | 0 | N/A |
| cgd2_3080 (TSP10) | 1176 | 4 | 0.00068 | -0.53321 |
| cgd5_3420 (TSP2) | 11610 | 13 | 0.00023 | -0.62332 |
| cgd6_780  (TSP8) | 2067 | 1 | 0.00003 | -1.147 |
| cgd5_4470 (TSP7) | 1971 | 2 | 0.00013 | -1.02235 |
| cgd6_800  (TSP9) | 1451 | 0 | 0 | N/A |

**Table S3.** Nucleotide and protein sequences for recombinant expression of CpTSP1_372-429_.

| Synthetic dsDNA sequence cloned into pET29 @ *NdeI/NotI* | AAAAA**CATATG**GGATCCTGGTCACATCCGCAGTTTGAAAA  AGGTAGCGAAACCTGTCTGGTTGGTAGCTGGTCAGAATGG  TCAGATTGTAGCACCAGCTGTGGTGAAGGTAATCGTATTC  GTACCCGTGAAATCACCAAACCGCCTCTGAATGGTGATGA  TAGCAAATGTCCGGAACTGATCGAAAAAGAAAGCTGCAAT  AAAGATGTGGAATGTCCGCATGGTAGCCATCACCATCATC  ATCATTAA**GCGGCCGC**AAAAA |
| --- | --- |
| CpTSP1_372-429_ sequence | MGS**WSHPQFEK**GSETCLVGSWSEWSDCSTSCGEGNRIRTR  EITKPPLNGDDSKCPELIEKESCNKDVECPHGS**HHHHHH** |

**
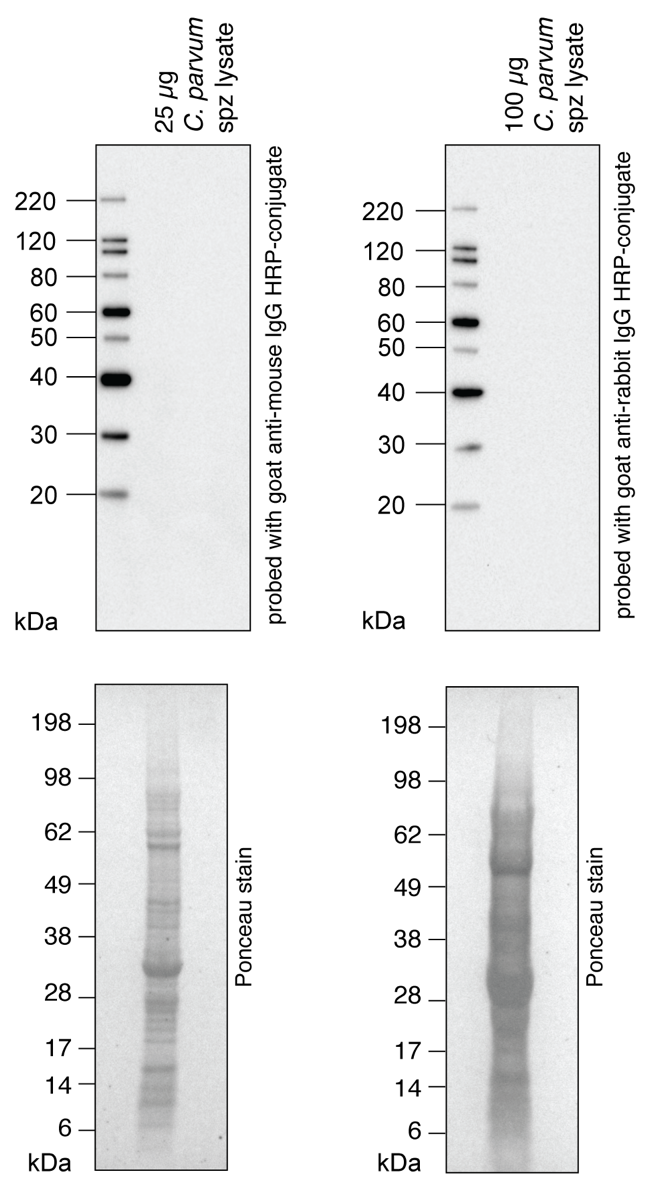
**

**Figure S1.** Western blots (and accompanying Ponceau-stained membrane) of *C. parvum* sporozoite lysate using secondary antibodies only to confirm the specificity of these reagents used in Figure 3A and 6A.


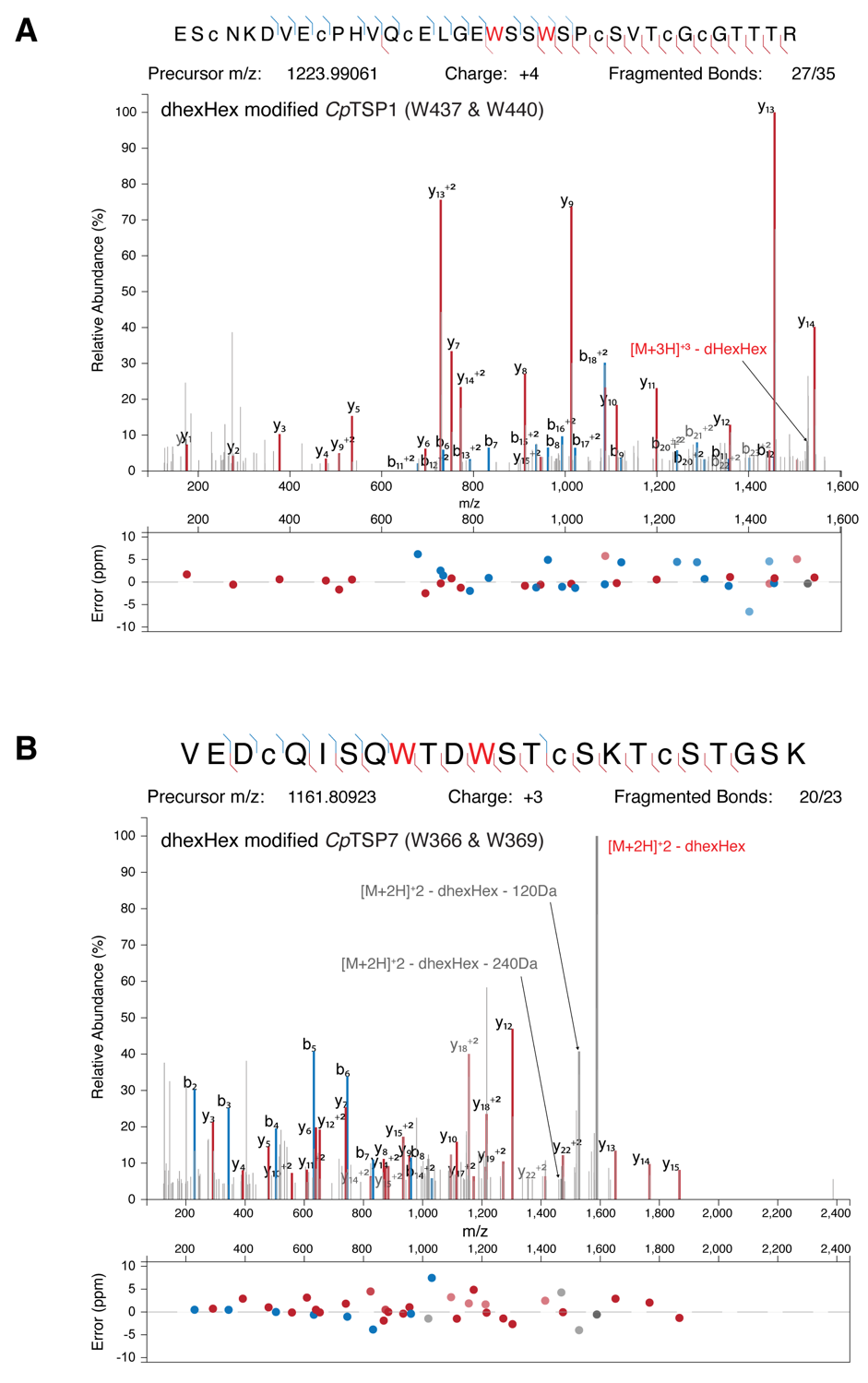


**Figure S2.** Tandem mass spectra for the C-Hex and O-dHexHex modified peptides from (A) *Cp*TSP1 and (B) *Cp*TSP7. A red ‘W’ indicates a Trp(Man) residue, while ‘c’ represents acetamidylcysteine. The site of O-dHexHex modification could not be identified due to its lability under the HCD technique.


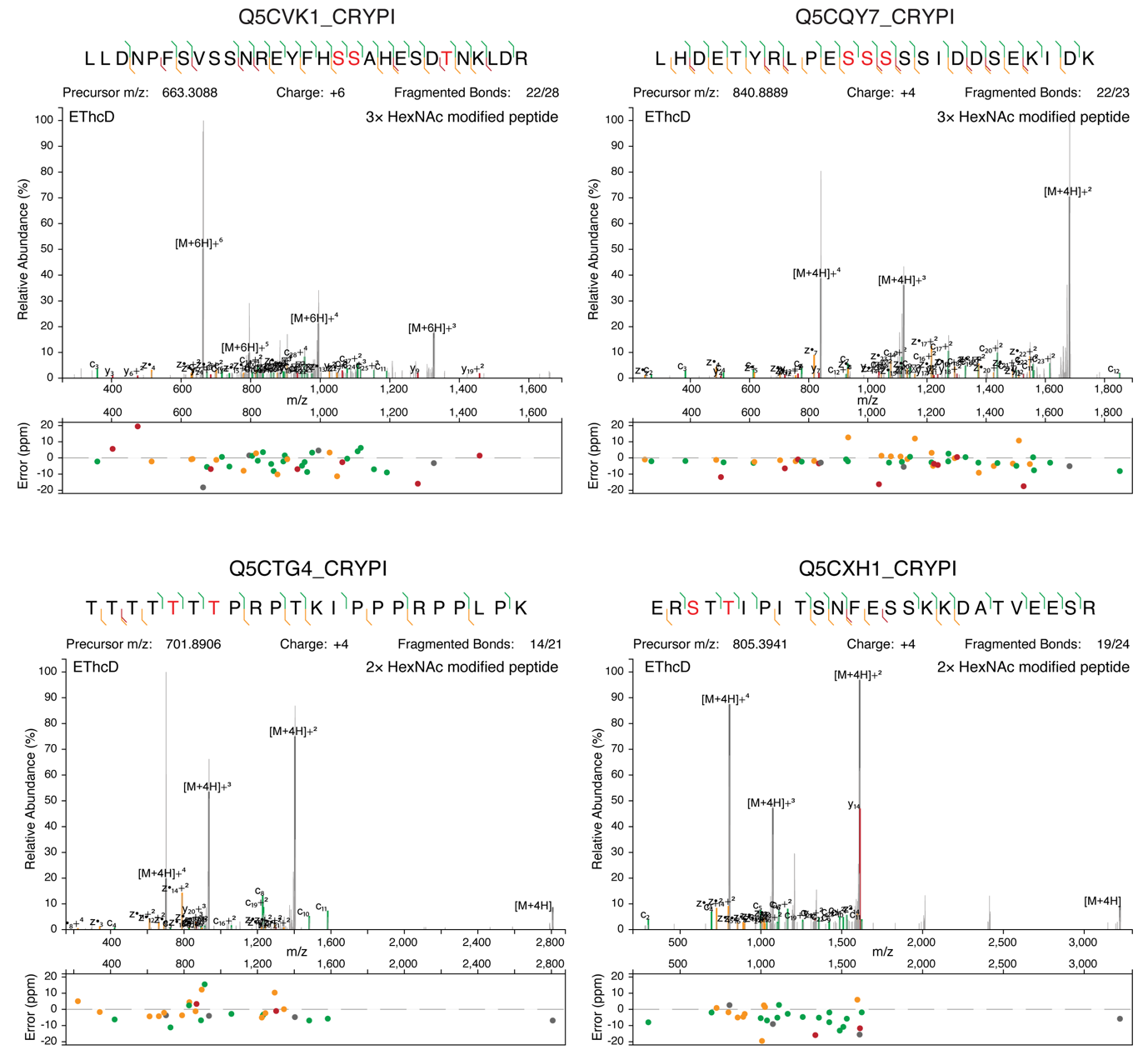


**Figure S3.** Annotated EThcD spectra of typical glycopeptides with multiple HexNAc modifications. Each HexNAc appears to be O-linked to a distinct Ser/Thr residue. The UniProt accession number is provided for each protein and the site of modification indicated by red text.
