# Supplementary material for: Conservation, abundance, glycosylation profile, and localization of the TSP protein family in Cryptosporidium parvum
Source: J Biol Chem. 2023 Feb 10;299(3):103006. doi: 10.1016/j.jbc.2023.103006 (PMC10034466; doi:10.1016/j.jbc.2023.103006)

Cryptosporidium parvum O-linked glycopeptides (manually confirmed)

Spectrum	Peptide	Charge	Delta Mass	Expectation	Hyperscore	Observed Modifications	Protein	Gene	Protein Description	Page
Nsco_1340_ZICHILIC_Crypto.49133.49133.3	TGKLIDPESGIAIDNSVSGVFATVPGTAAPK	3	203.0748	0	55.61	HexNAc(203.079373)	tr|Q5CY21|Q5CY21_CRYPI	cgd7_4020	Cryptopsoridial mucin, large thr stretch, signal peptide sequence	2
Nsco_1340_ZICHILIC_Crypto.22945.22945.3	IKVNGQDFSTLSANSSSPTENGGSAGQASSR	3	203.0777	0	70.964	HexNAc(203.079373)	tr|Q5CXH4|Q5CXH4_CRYPI	cgd6_1080	GP40 domain-containing protein	3
Nsco_1340_ZICHILIC_Crypto.41607.41607.4	SGSTTSSTGSNTKDEEIAITFEKPEIQEVSTQVENLTKPQETNTSYK	4	203.0798	4.05505E-08	29.464	HexNAc(203.079373)	tr|Q5CXH9|Q5CXH9_CRYPI	cgd6_1030	Uncharacterized protein	4
Nsco_1340_ZICHILIC_Crypto.21364.21364.2	VNGQDFSTLSANSSSPTENGGSAGQASSR	2	203.0804	0	61.989	HexNAc(203.079373)	tr|Q5CXH4|Q5CXH4_CRYPI	cgd6_1080	GP40 domain-containing protein	5
Nsco_1340_ZICHILIC_Crypto.04841.04841.3	HAKPTASASSYTPQKHHPTVSVNSEKK	3	203.0836	6.4E-13	34.056	HexNAc(203.079373)	tr|Q5CRM5|Q5CRM5_CRYPI	cgd5_2230	Membrane associated thioredoxin	6
Nsco_1340_ZICHILIC_Crypto.19993.19993.2	TSSAQKDEESFSDSTVATEGDEGSEEEQE	2	203.0838	0	64.891	HexNAc(203.079373)	tr|Q5CXZ8|Q5CXZ8_CRYPI	cgd7_4280	Signal peptide, large secreted protein	7
Nsco_1340_ZICHILIC_Crypto.05896.05896.5	HAKPTASASSYTPQKHHPTVSVNSEK	5	203.0857	0	57.876	HexNAc(203.079373)	tr|Q5CRM5|Q5CRM5_CRYPI	cgd5_2230	Membrane associated thioredoxin	8
Nsco_1340_ZICHILIC_Crypto.31326.31326.3	NSESEPEPASVASSENESQMTKPDQGIEPTVEESSTEIR	3	203.0891	0	76.637	HexNAc(203.079373)	tr|Q5CTG6|Q5CTG6_CRYPI	cgd2_3090	Uncharacterized protein	9
Nsco_1340_ZICHILIC_Crypto.09183.09183.4	NANGIQTAISSKHTAGTHHYSTPSAECVR	4	203.0893	0.000149499	23.486	HexNAc(203.079373)	tr|Q5CX85|Q5CX85_CRYPI	cgd6_2090	CpCOWP1, oocyst wall protein with type I and type II cysteine-rich repeats (Fragment)	10
Nsco_1340_ZICHILIC_Crypto.28200.28200.5	KIGSDAQHTEPFQPLLDASEEKQDEKDSIIDAAK	5	406.1592	5.6E-13	40.863	HexNAc(2)(406.158745)	tr|Q5CXH1|Q5CXH1_CRYPI	cgd6_1110	Signal peptide-containing protein	11
Nsco_1340_ZICHILIC_Crypto.14865.14865.4	ERSTTIPITSNFESSKKDATVEESR	4	406.1602	7.7E-13	39.353	HexNAc(2)(406.158745)	tr|Q5CS78|Q5CS78_CRYPI	cgd1_3780	Domain KOG1015, transcription regulator XNP/ATRX, DEAD-box superfamily, signal peptide	12
Nsco_1340_ZICHILIC_Crypto.13529.13529.5	RLHDETYRLPESSSSSIDDSEKIDK	5	406.161	1.694E-11	39.211	HexNAc(2)(406.158745)	tr|Q5CQY7|Q5CQY7_CRYPI	cgd4_2800	LITAF domain-containing protein	13
Nsco_1340_ZICHILIC_Crypto.44988.44988.3	AFTRNPVAYNQSIQSGTTPAPTPAPTPAPTPIPAPTPVPLPR	3	406.1629	0	72.715	HexNAc(2)(406.158745)	tr|Q5CTG4|Q5CTG4_CRYPI	cgd2_3110	Uncharacterized protein	14
Nsco_1340_ZICHILIC_Crypto.12521.12521.4	NVVVQPTMASQQQEIIQTTITGGRHHHH	4	406.1632	0	46.875	HexNAc(2)(406.158745)	tr|Q5CPJ0|Q5CPJ0_CRYPI	cgd6_200	Oocyst wall protein 8	15
Nsco_1340_ZICHILIC_Crypto.11394.11394.4	TTTTTTTPRPTKIPPPRPPLPK	4	406.1677	1.349E-11	37.69	HexNAc(2)(406.158745)	tr|Q5CTG4|Q5CTG4_CRYPI	cgd2_3110	Uncharacterized protein	16
Nsco_1340_ZICHILIC_Crypto.37425.37425.2	DVSISAIATDTTTITTTAMPTSSKFQK	2	609.2283	0	48.871	HexNAc(3)(609.238118)	tr|Q5CQS3|Q5CQS3_CRYPI	cgd4_3530	Uncharacterized protein	17
Nsco_1340_ZICHILIC_Crypto.41280.41280.3	TQSTTAPPSPFPPPSPPPSPLPPPLPPKVR	3	609.2298	0	62.442	HexNAc(3)(609.238118)	tr|Q5CV53|Q5CV53_CRYPI	cgd8_5380	Uncharacterized protein	18
Nsco_1340_ZICHILIC_Crypto.38914.38914.2	DVSISAIATDTTTITTTAMPTSSK	2	609.2382	0	62.445	HexNAc(3)(609.238118)	tr|Q5CQS3|Q5CQS3_CRYPI	cgd4_3530	Uncharacterized protein	19
Nsco_1340_ZICHILIC_Crypto.15304.15304.4	LHDETYRLPESSSSSIDDSEKIDK	4	609.2396	0	51.359	HexNAc(3)(609.238118)	tr|Q5CQY7|Q5CQY7_CRYPI	cgd4_2800	LITAF domain-containing protein	20
Nsco_1340_ZICHILIC_Crypto.39150.39150.3	DVSISAIATDTTTITTTAMPTSSKFQKFDITK	3	609.2417	0	67.039	HexNAc(3)(609.238118)	tr|Q5CQS3|Q5CQS3_CRYPI	cgd4_3530	Uncharacterized protein	21
Nsco_1340_ZICHILIC_Crypto.22391.22391.6	LLDNPFSVSSNREYFHSSAHESDTNKLDR	6	609.2424	1.1199E-10	30.184	HexNAc(3)(609.238118)	tr|Q5CVK1|Q5CVK1_CRYPI	cgd8_3760	Predicted membrane associated protein, signal peptide, transmembrane domain near C	22
Nsco_1340_ZICHILIC_Crypto.46564.46564.3	TQSTTAPPSPFPPPSPPPSPLPPPLPPK	3	609.2467	1.07662E-05	24.826	HexNAc(3)(609.238118)	tr|Q5CV53|Q5CV53_CRYPI	cgd8_5380	Uncharacterized protein	23
Nsco_1340_ZICHILIC_Crypto.07088.07088.5	SSTTTTTTTAPVSSEDNKPEDSEDEKESESESDSSNEGSSNSEDSEEKTR	5	609.2472	0	48.8	HexNAc(3)(609.238118)	tr|Q5CYR7|Q5CYR7_CRYPI	cgd7_1280	Uncharacterized protein	24

Q5CY21_CRYPI-Cryptopsoridial mucin, large thr stretch, signal peptide sequence

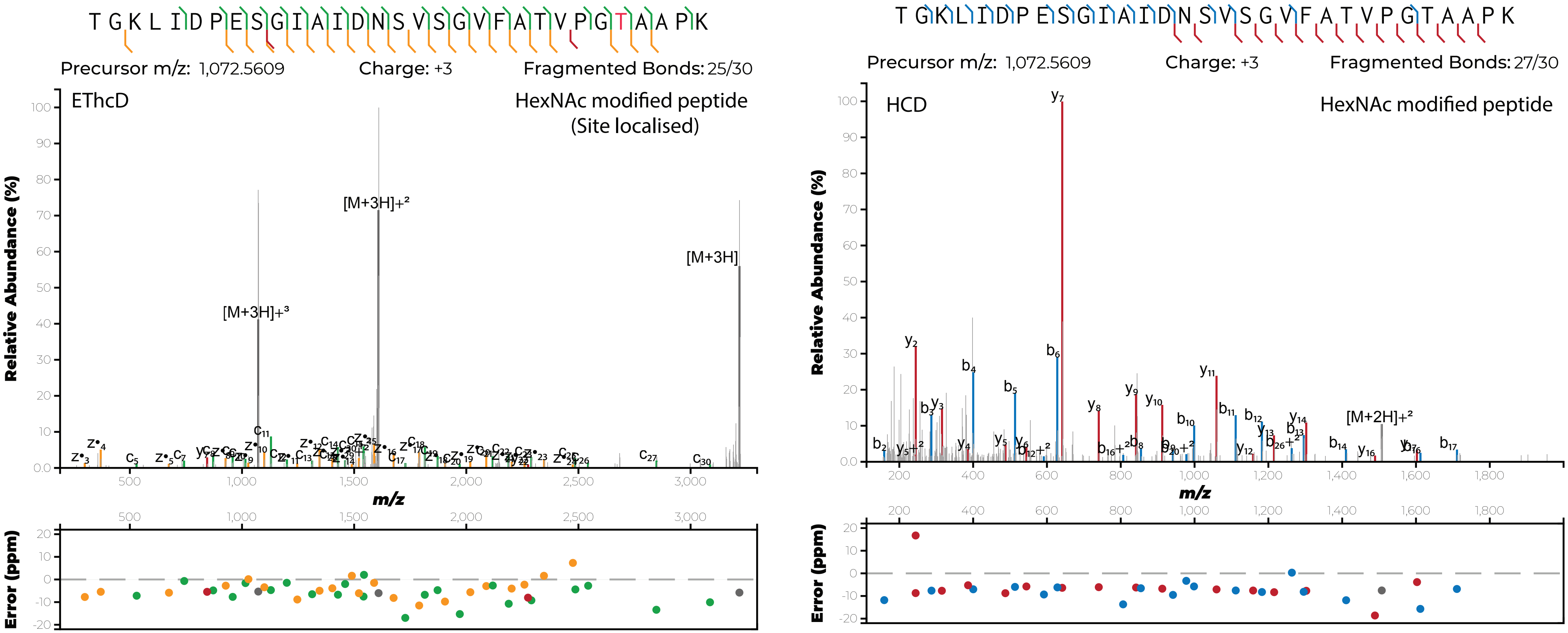

Q5CXH4_CRYPI - GP40 domain-containing protein

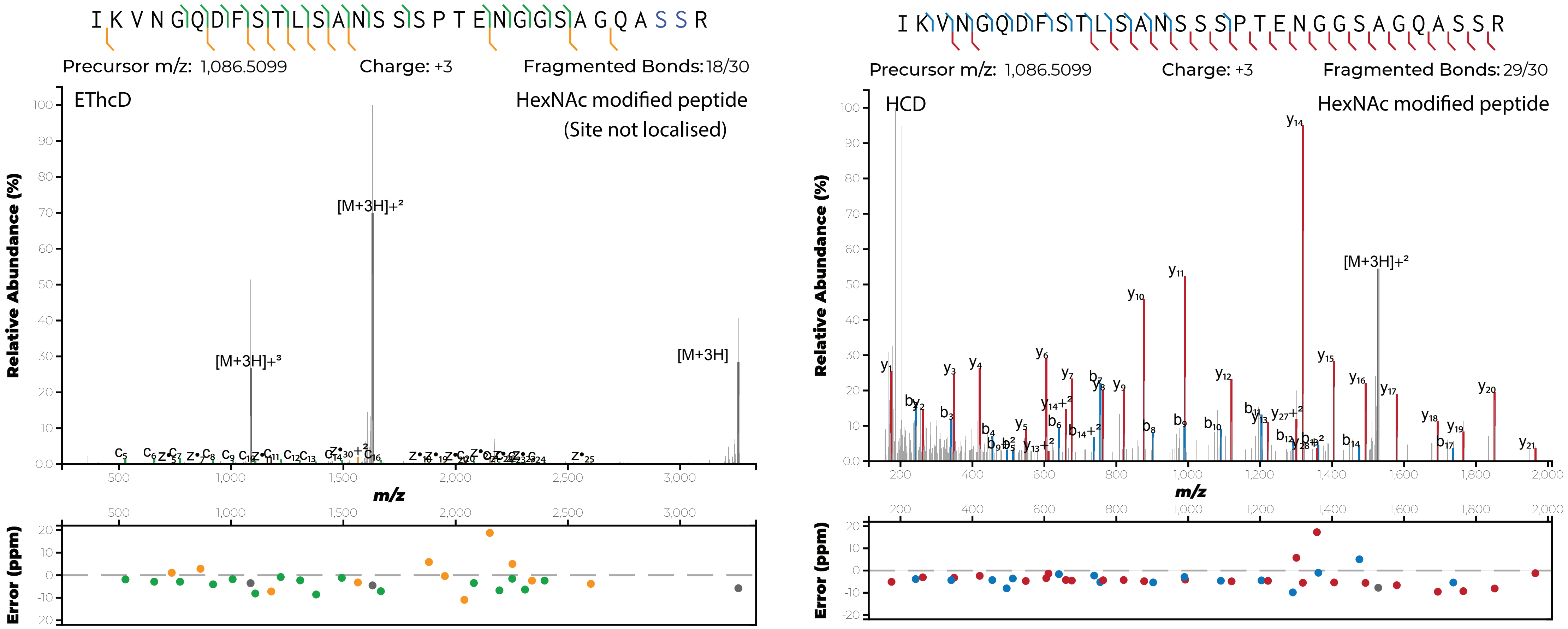

Q5CXH9_CRYPI - Uncharacterized protein

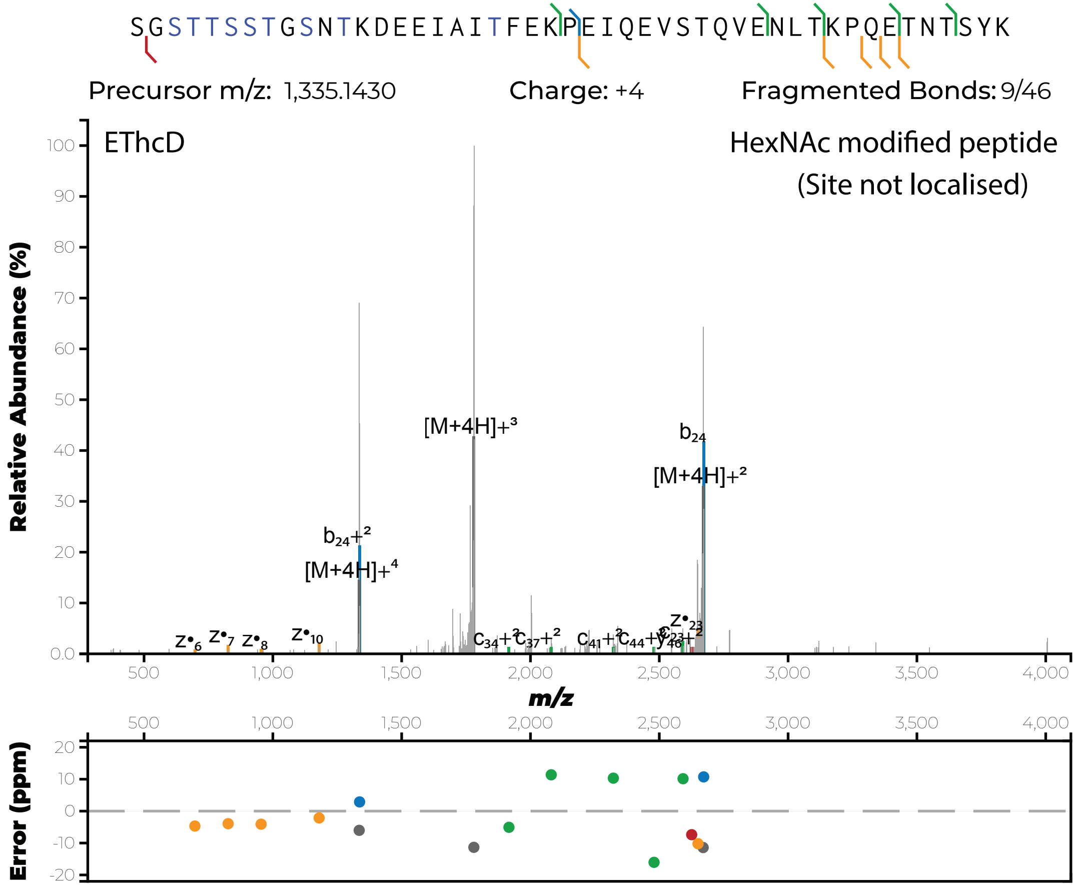

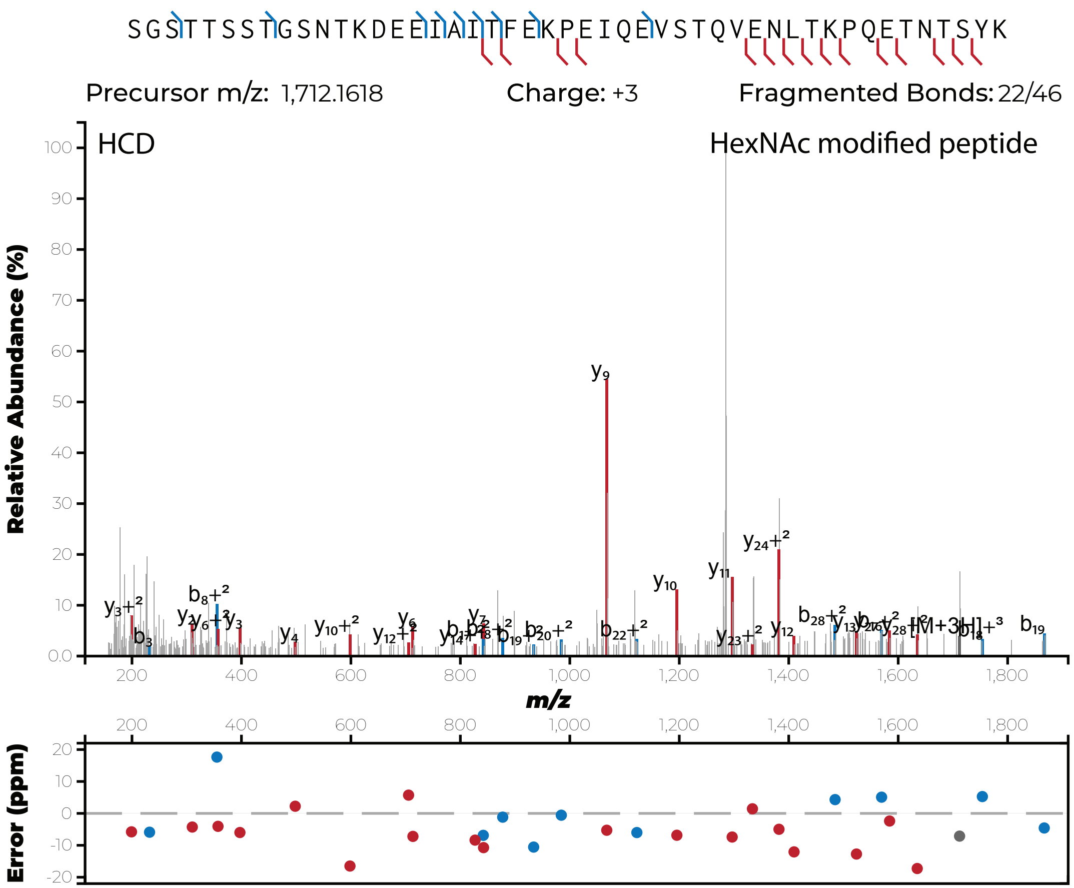

Q5CXH4_CRYPI-GP40 domain-containing protein

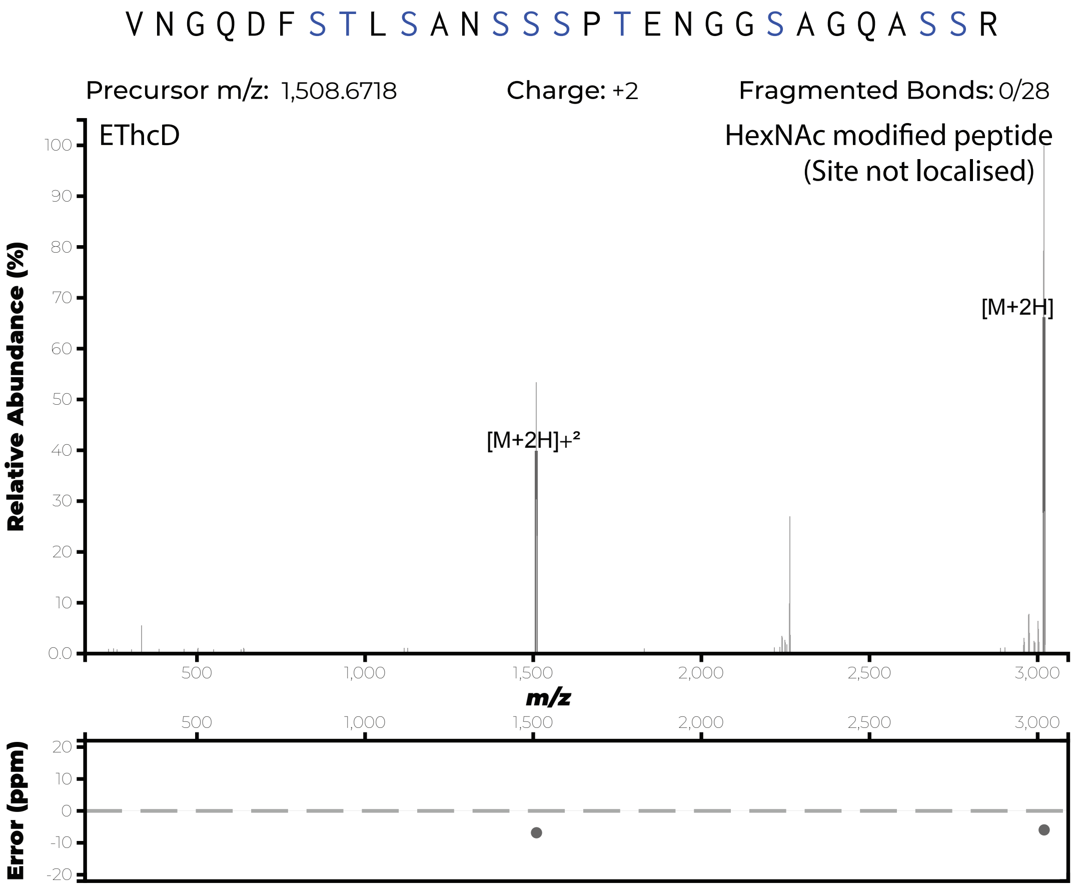

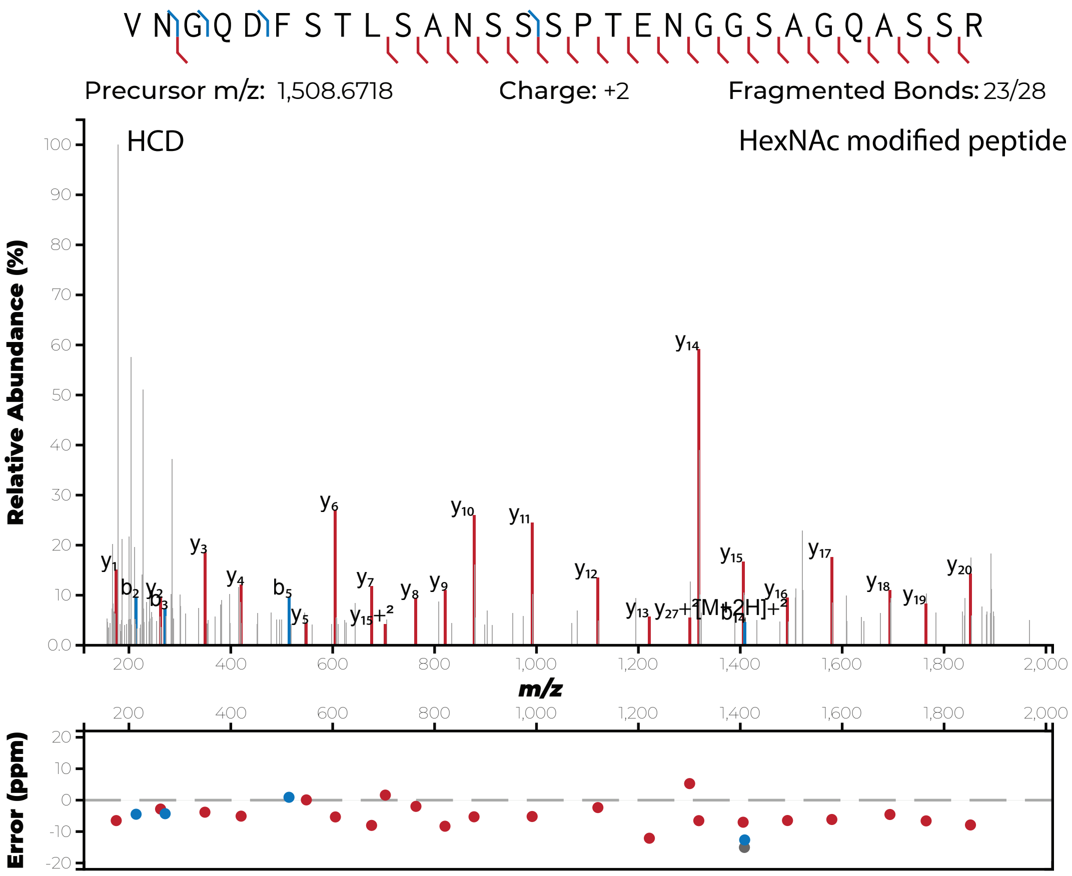

Q5CXH4_CRYPI-GP40 domain-containing protein

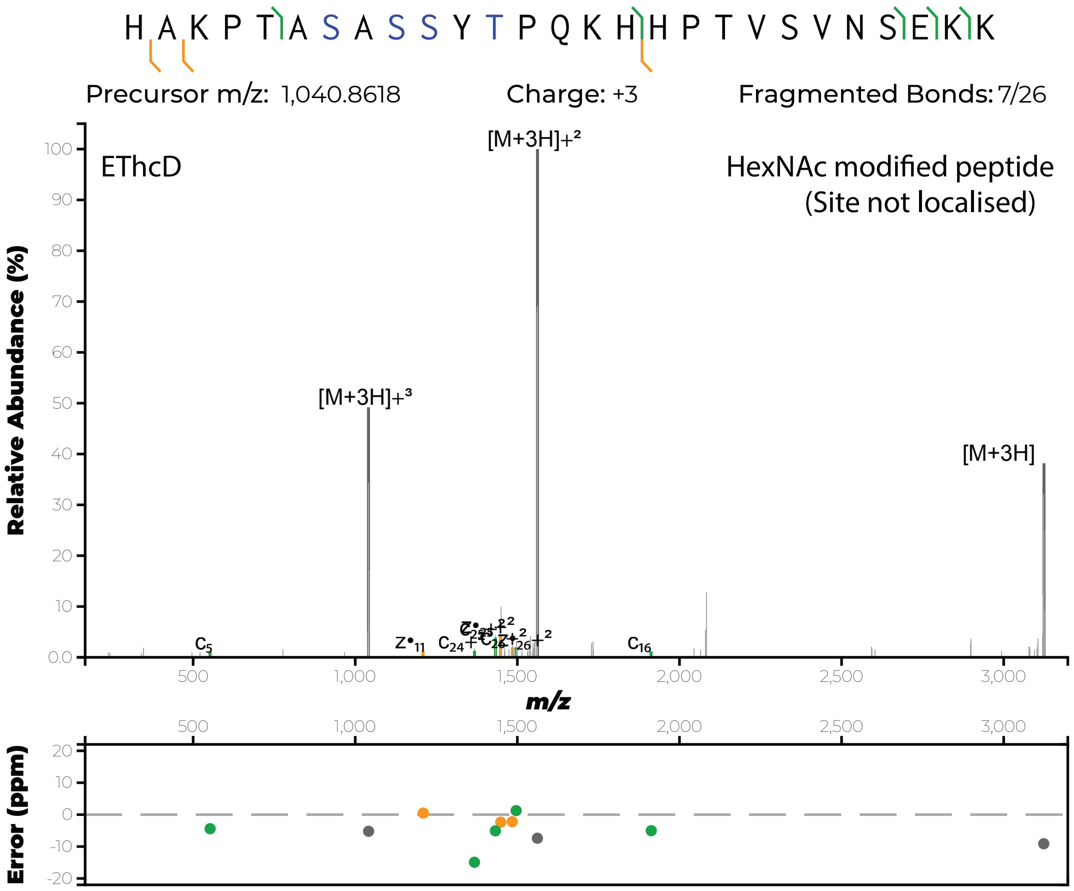

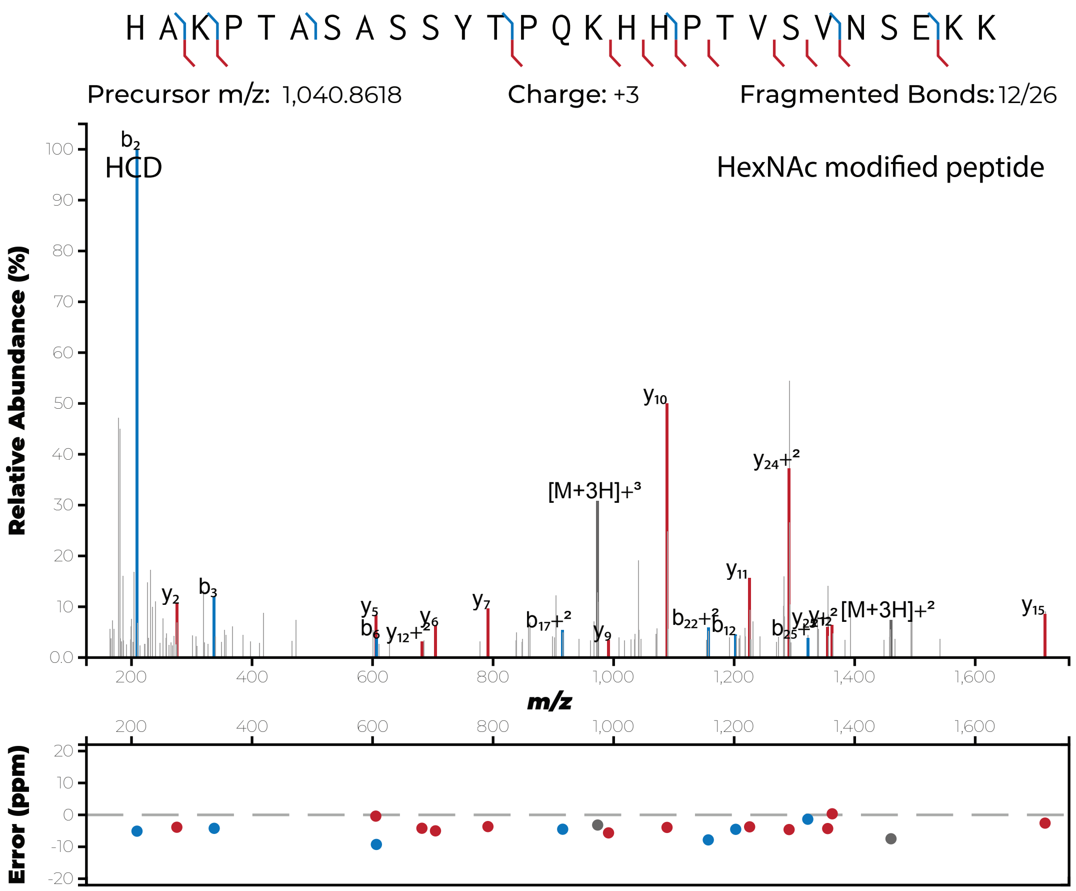

Q5CXZ8_CRYPI-Signal peptide, large secreted protein

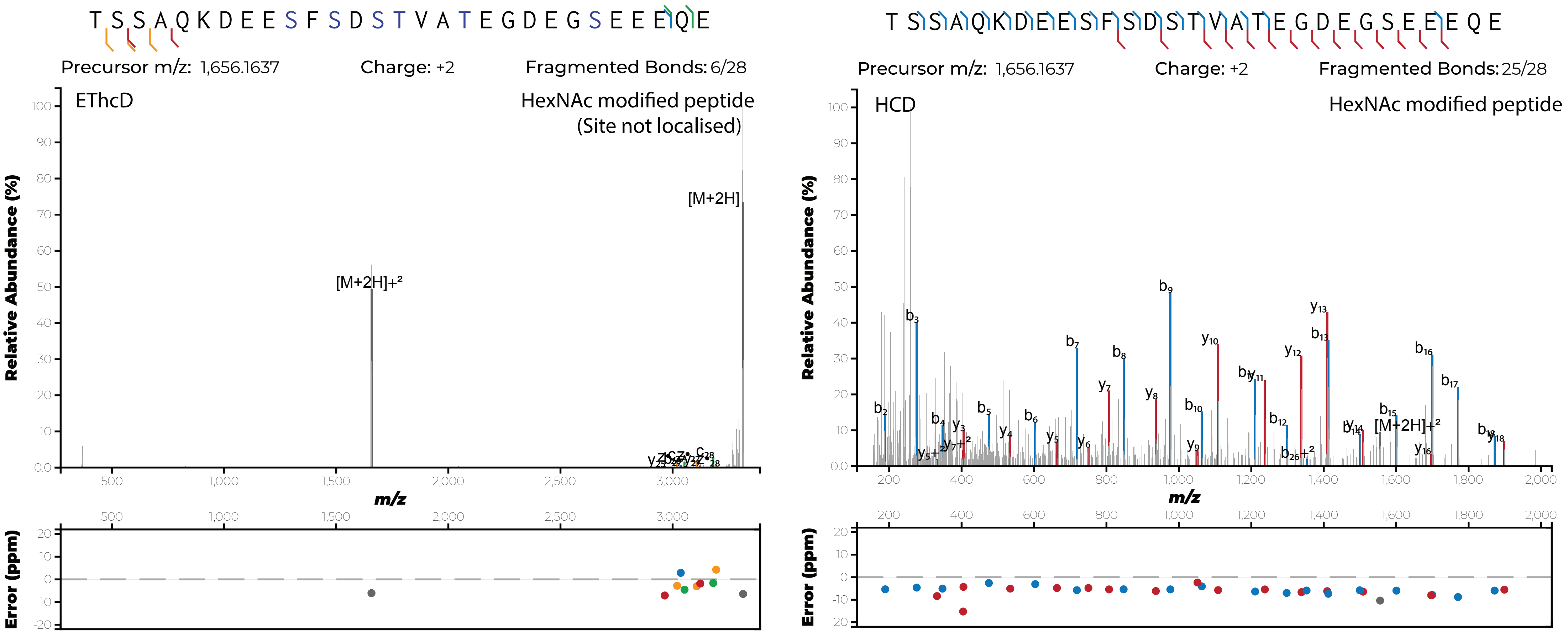

Q5CRM5_CRYPI-Membrane associated thioredoxin

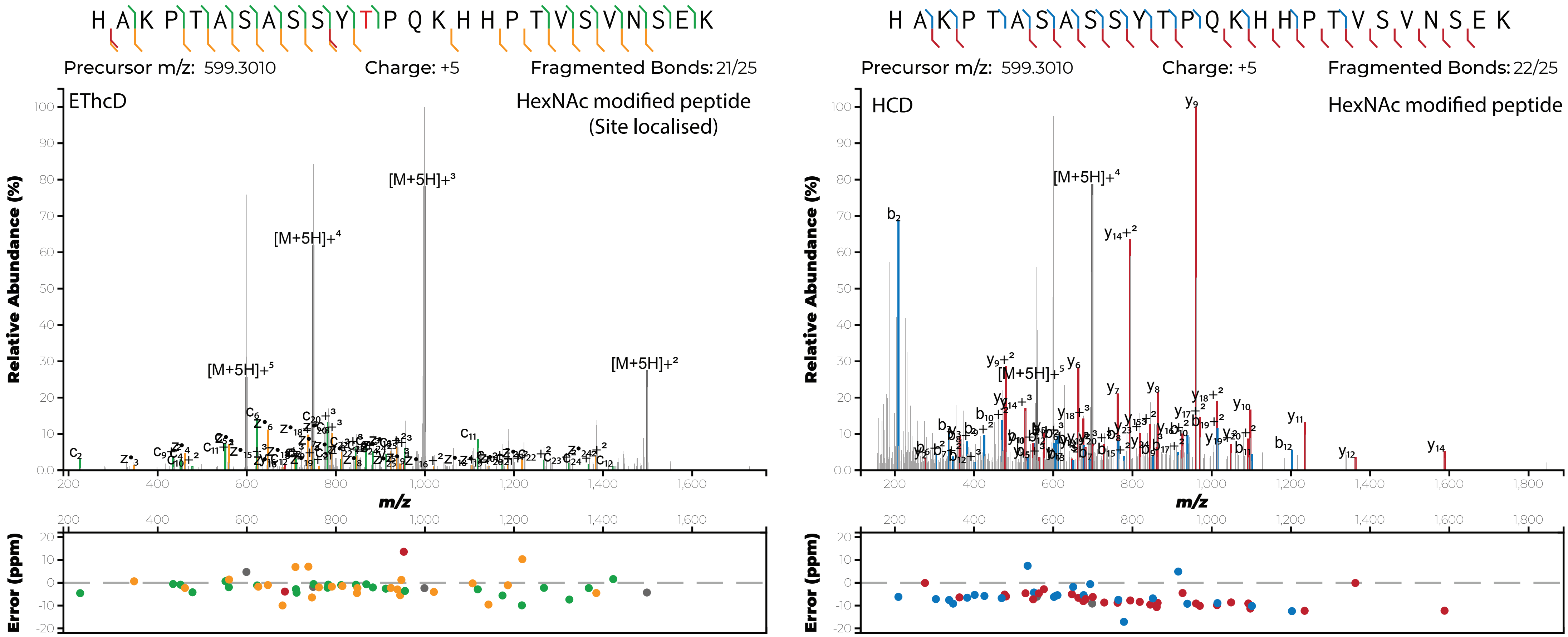

Q5CRM5_CRYPI-Membrane associated thioredoxin

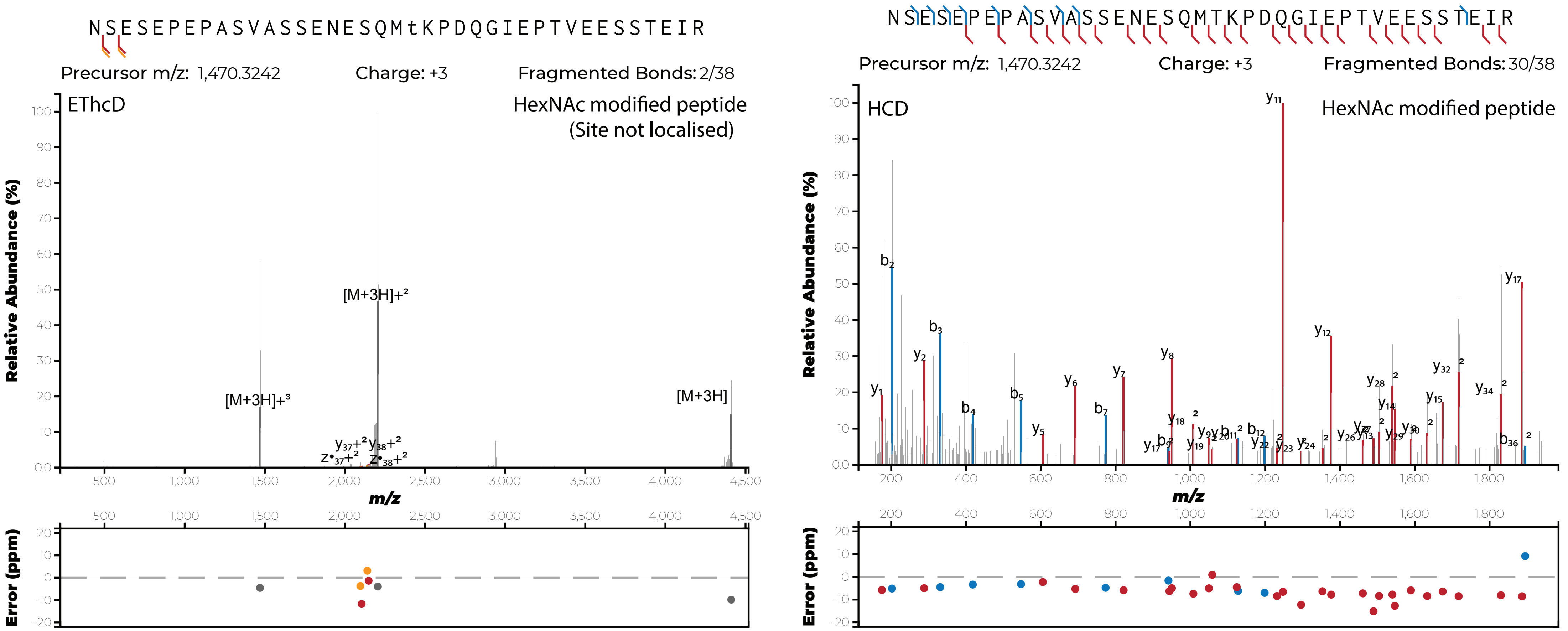

Q5CX85_CRYPI-CpCOWP1, oocyst wall protein with type I and type II cysteine-rich repeats

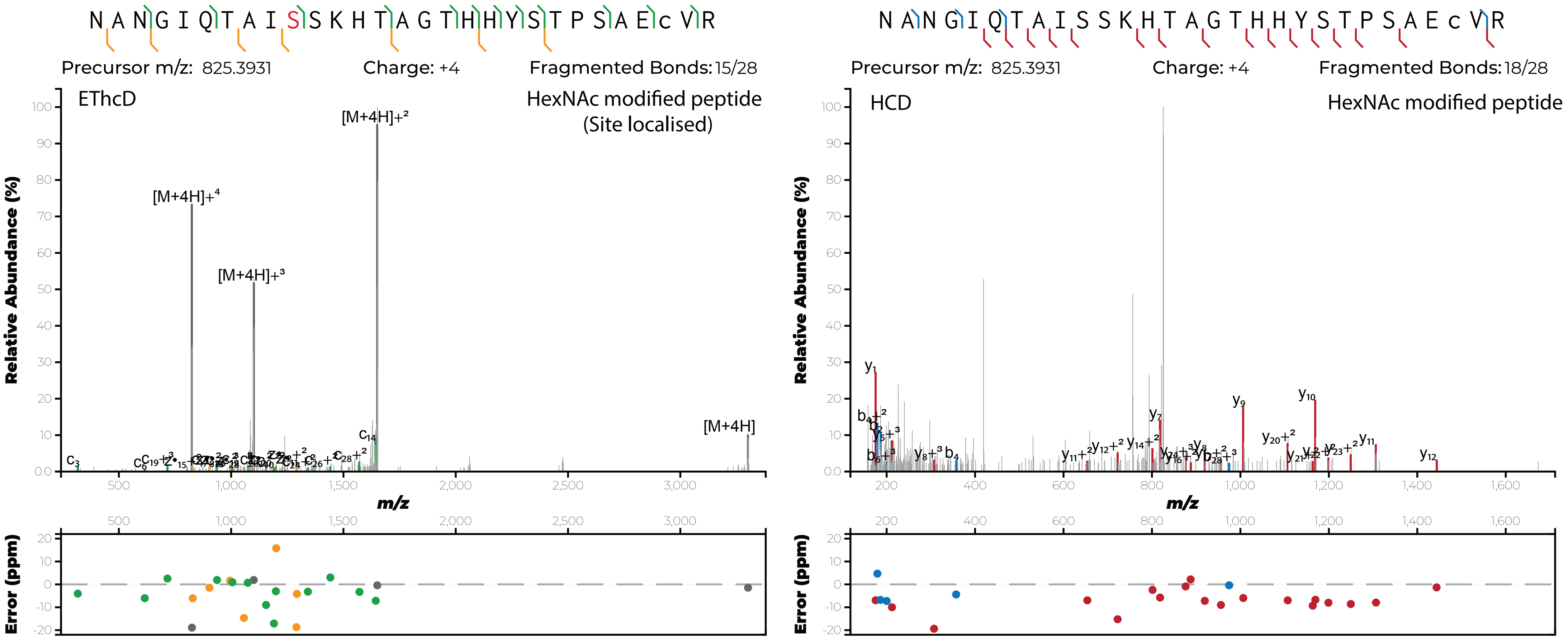

Q5CXH1_CRYPI-Signal peptide-containing protein

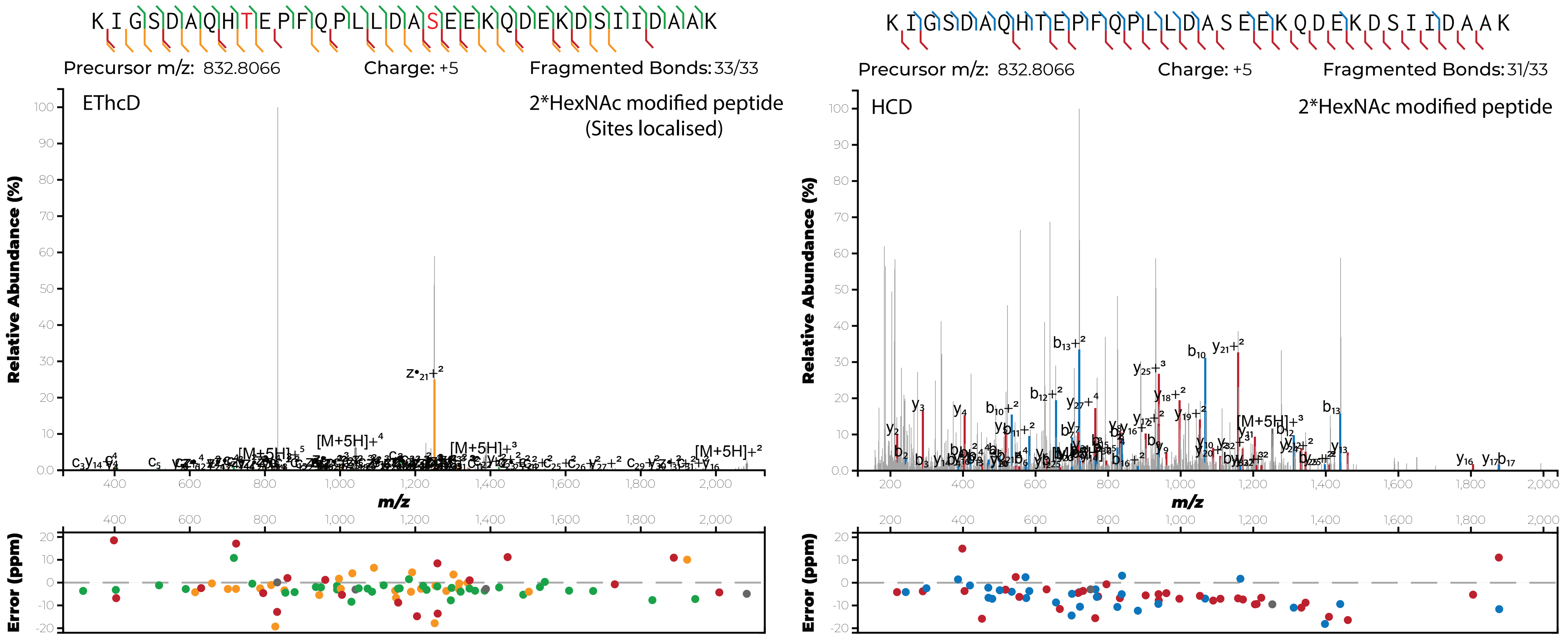

Q5CXH1_CRYPI-Signal peptide-containing protein

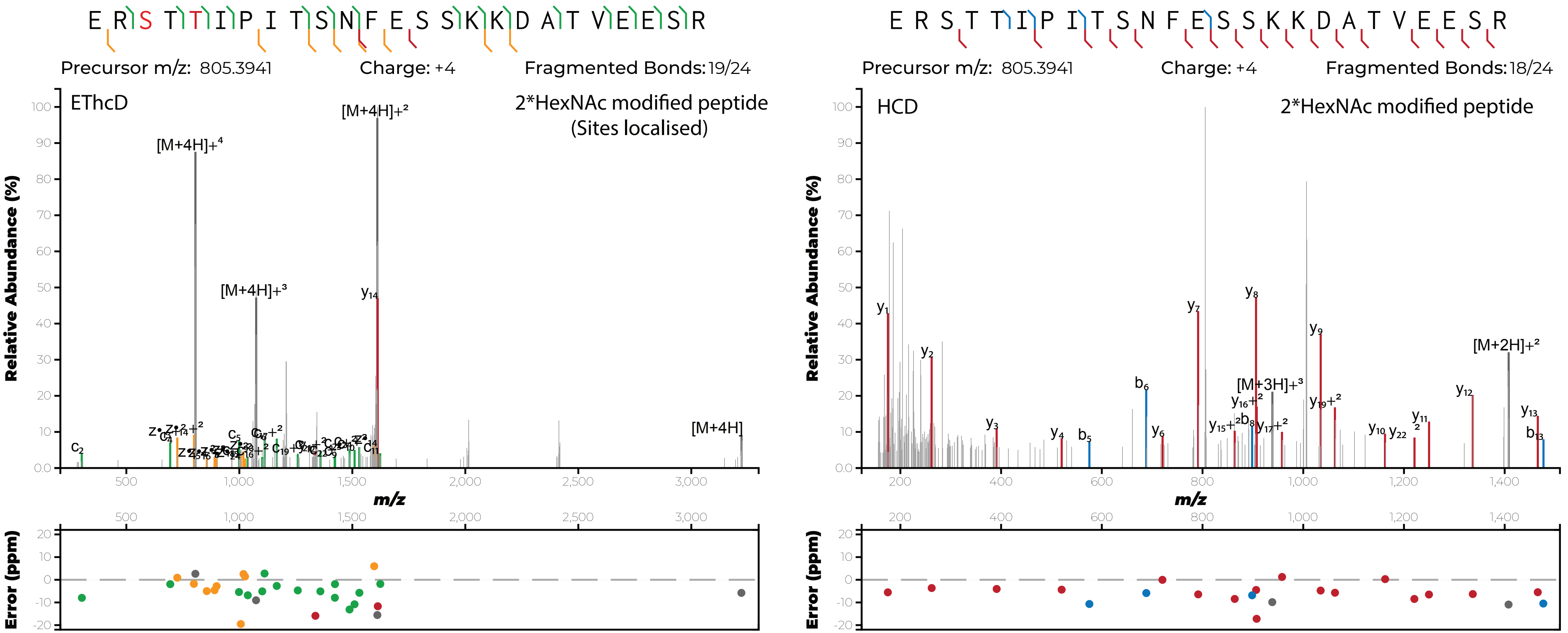

Q5CQY7_CRYPI-LITAF domain-containing protein

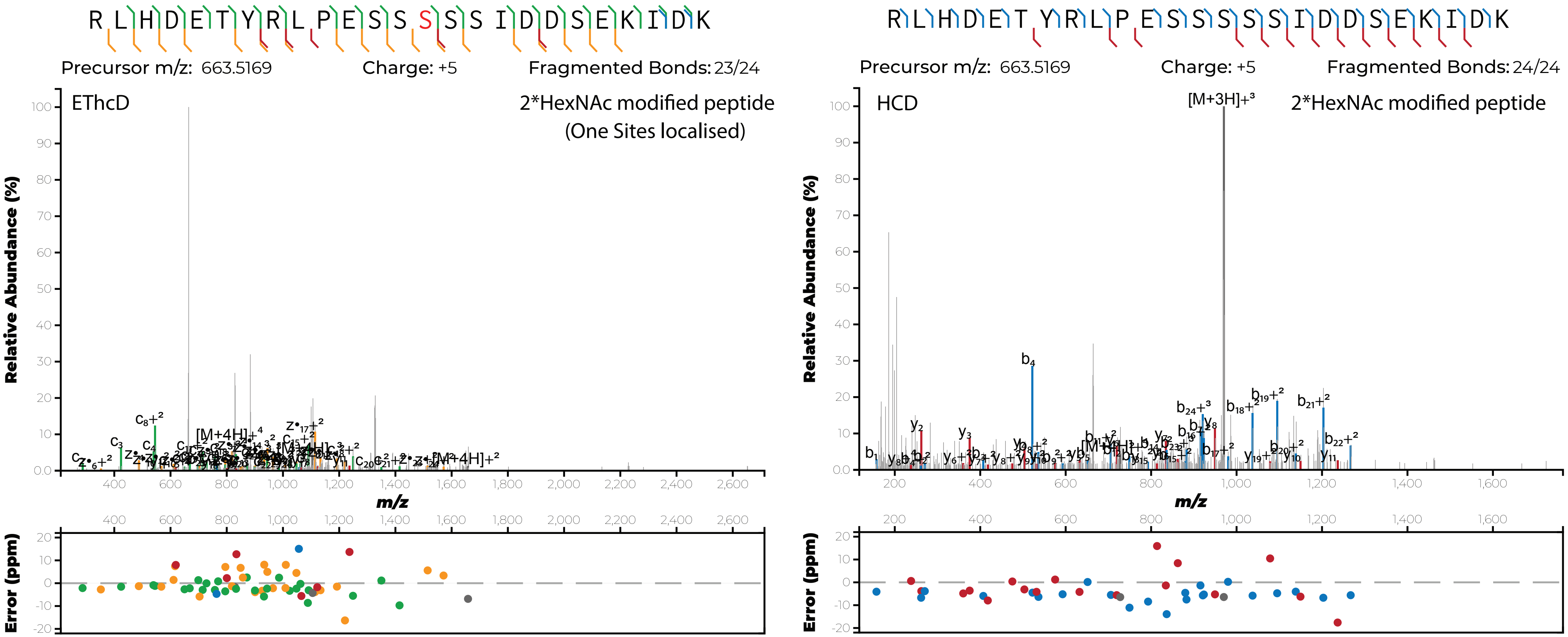

Q5CTG4_CRYPI-Uncharacterized protein

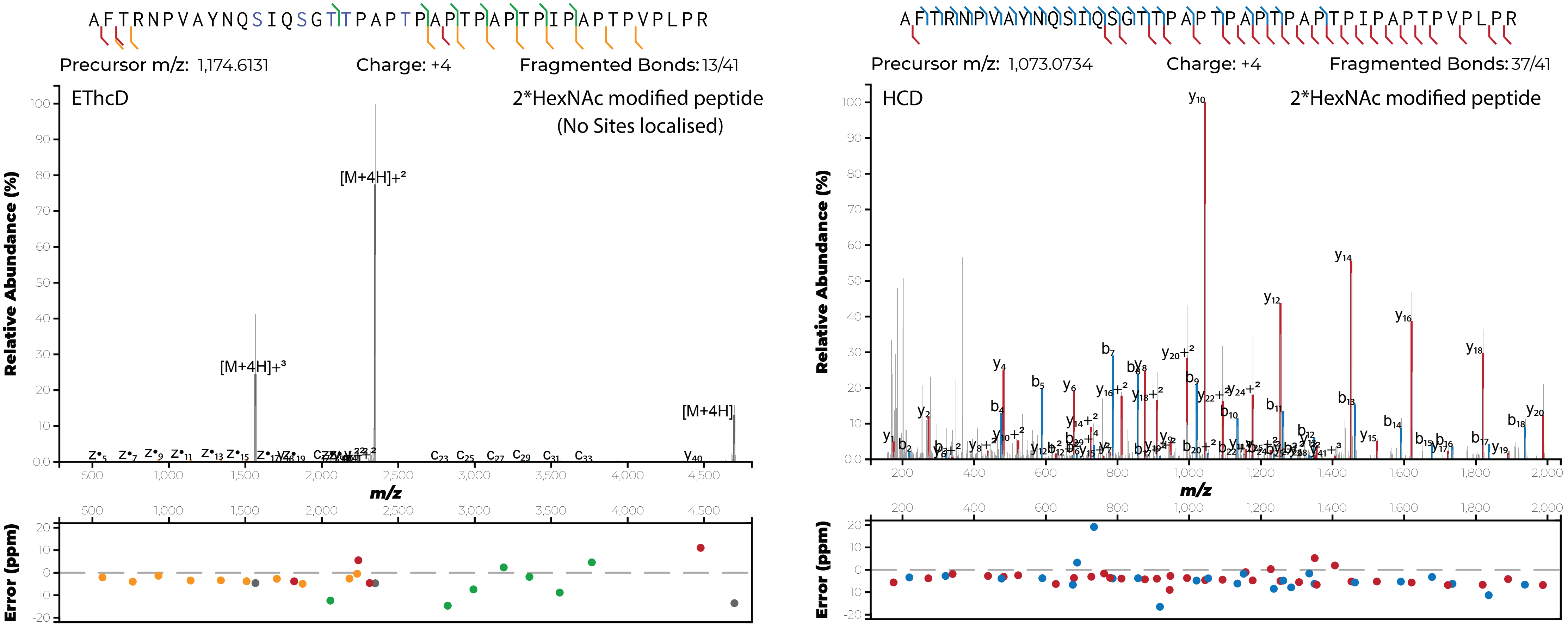

Q5CPJ0_CRYPI-Oocyst wall protein 8

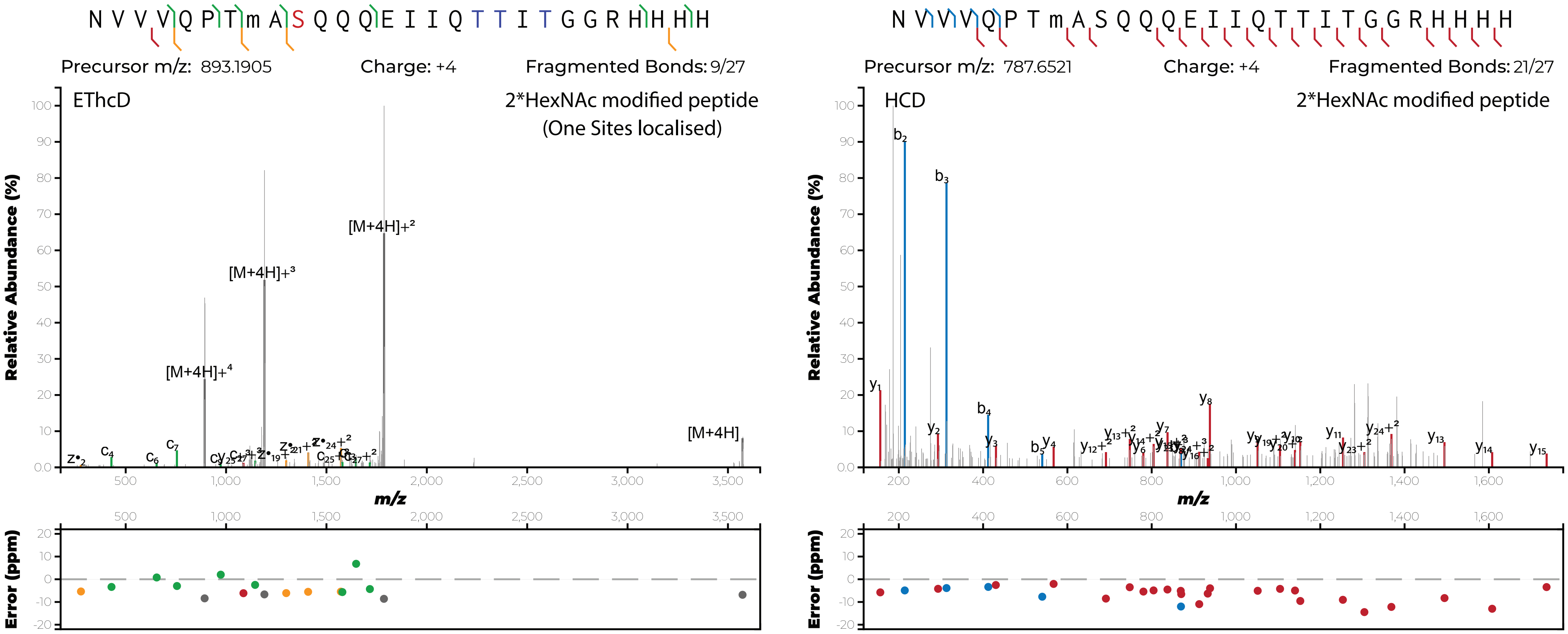

Q5CTG4_CRYPI-Uncharacterized protein

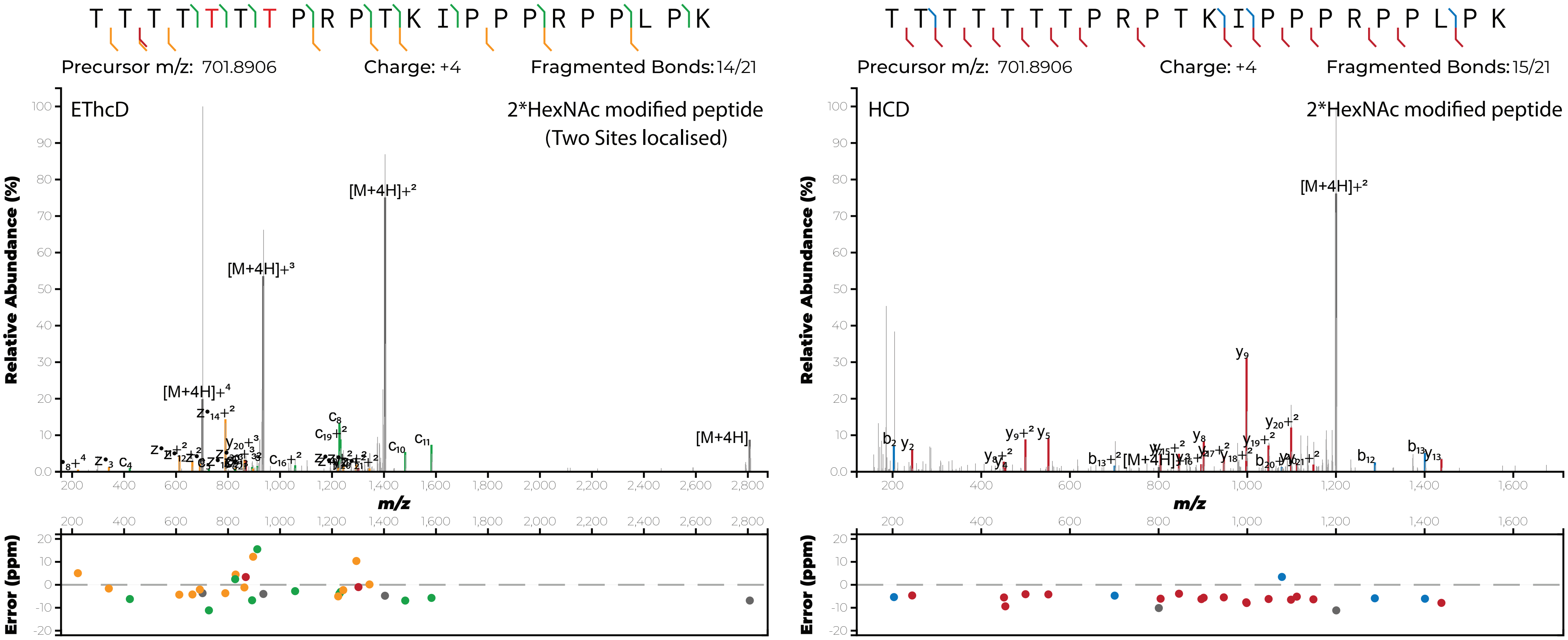

Q5CQS3_CRYPI-Uncharacterized protein

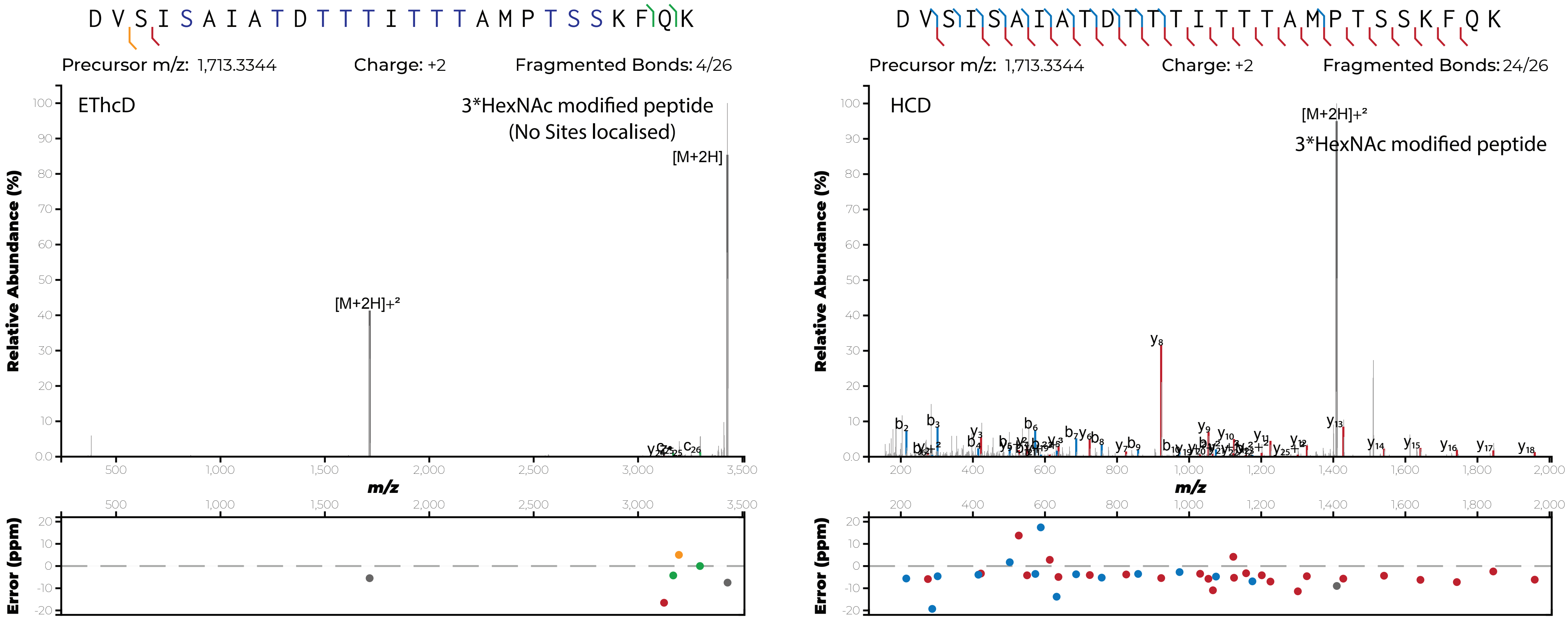

Q5CV53_CRYPI-Uncharacterized protein

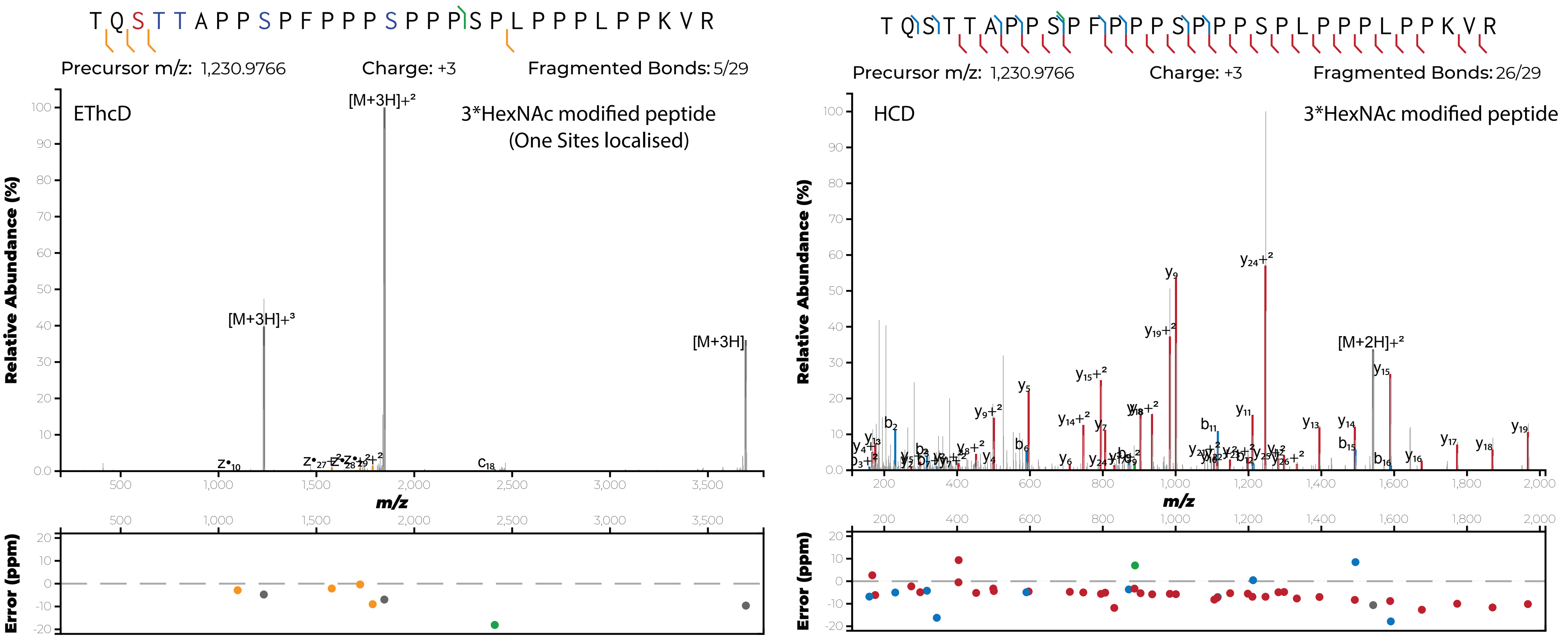

Q5CQS3_CRYPI-Uncharacterized protein

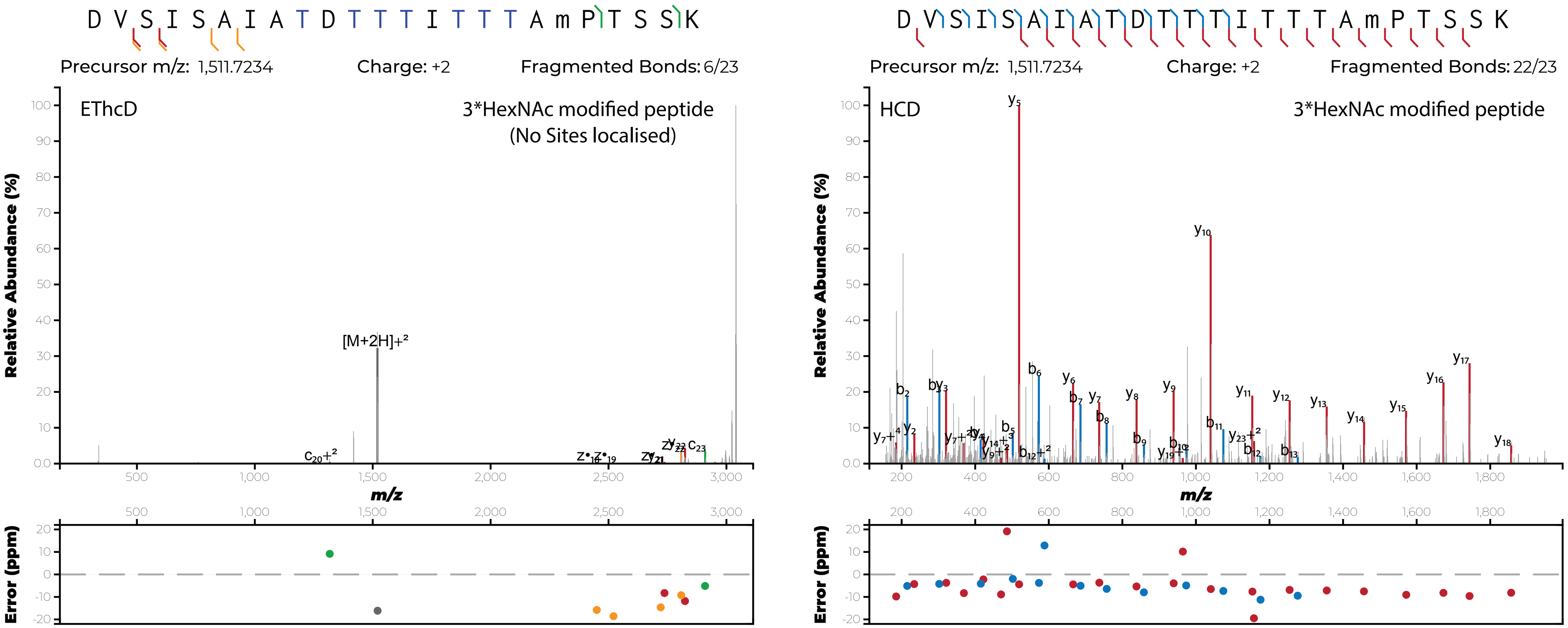

Q5CQY7_CRYPI-LITAF domain-containing protein

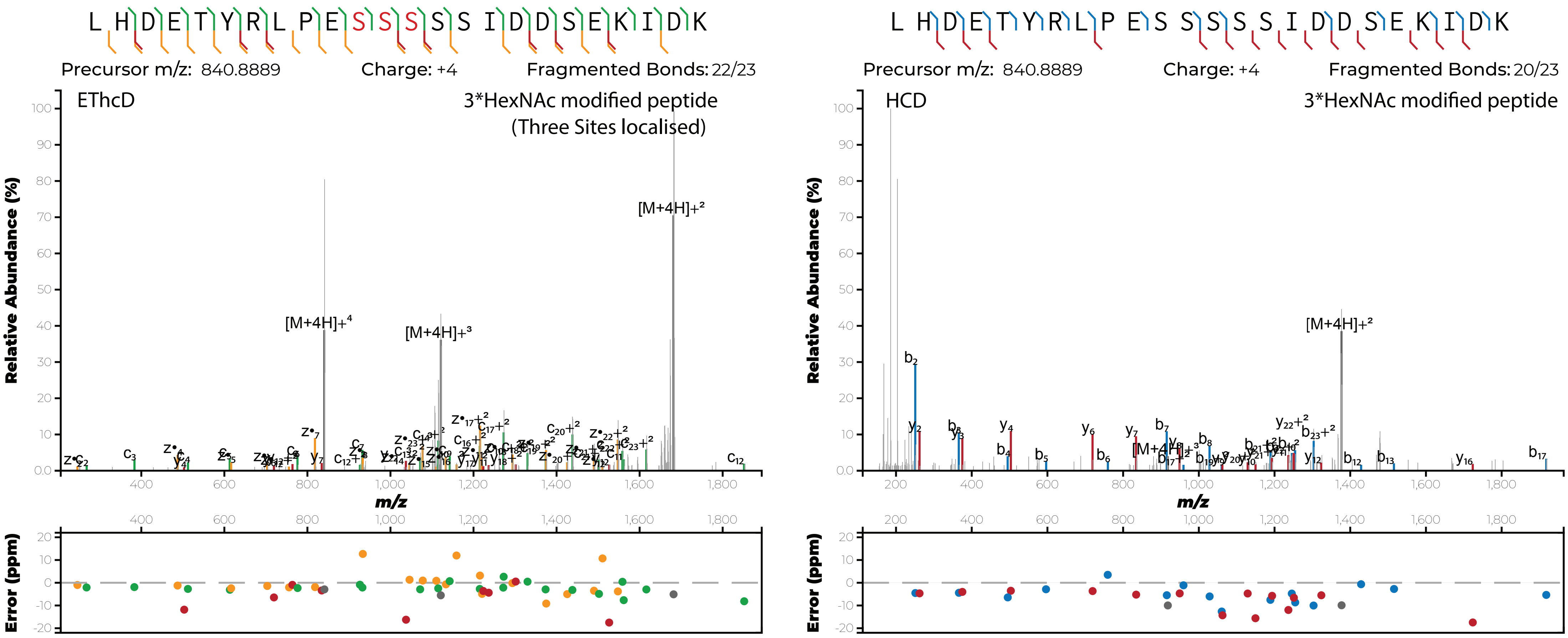

Q5CQY7_CRYPI-LITAF domain-containing protein

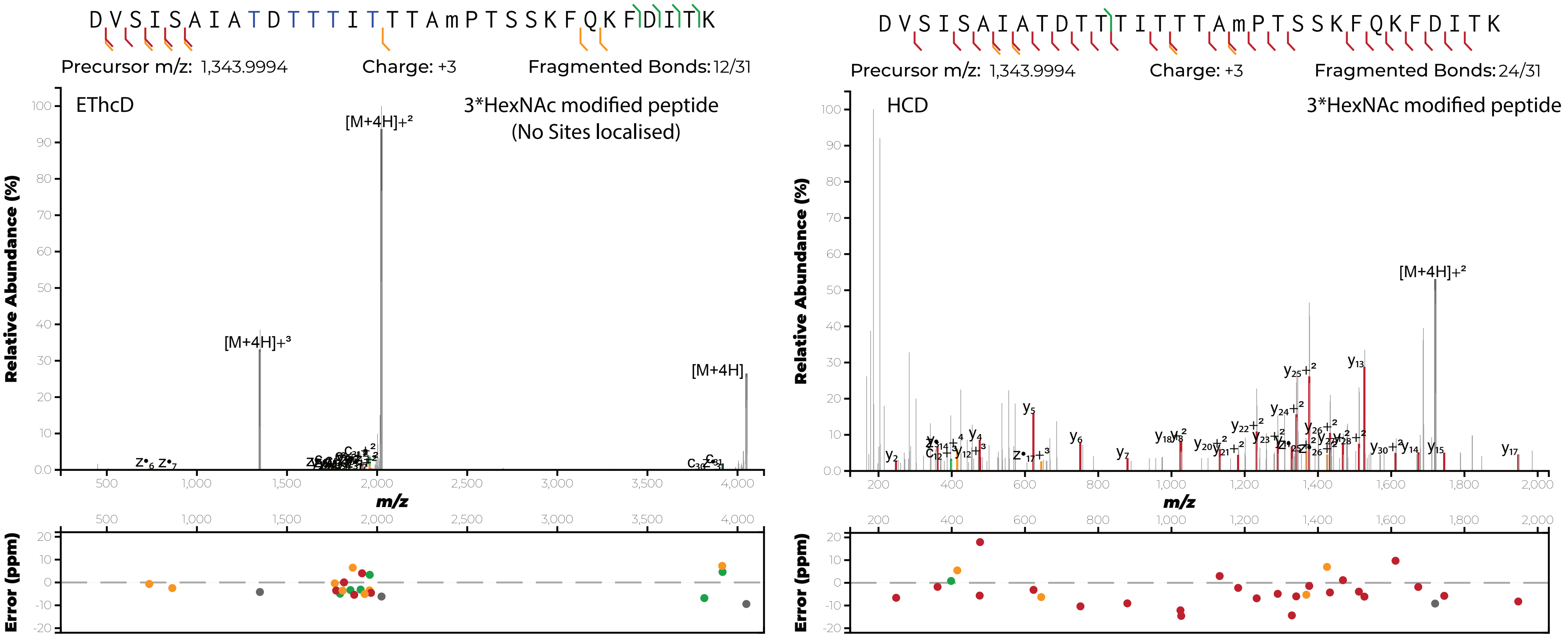

Q5CVK1_CRYPI-Predicted membrane associated protein, signal peptide, transmembrane domain

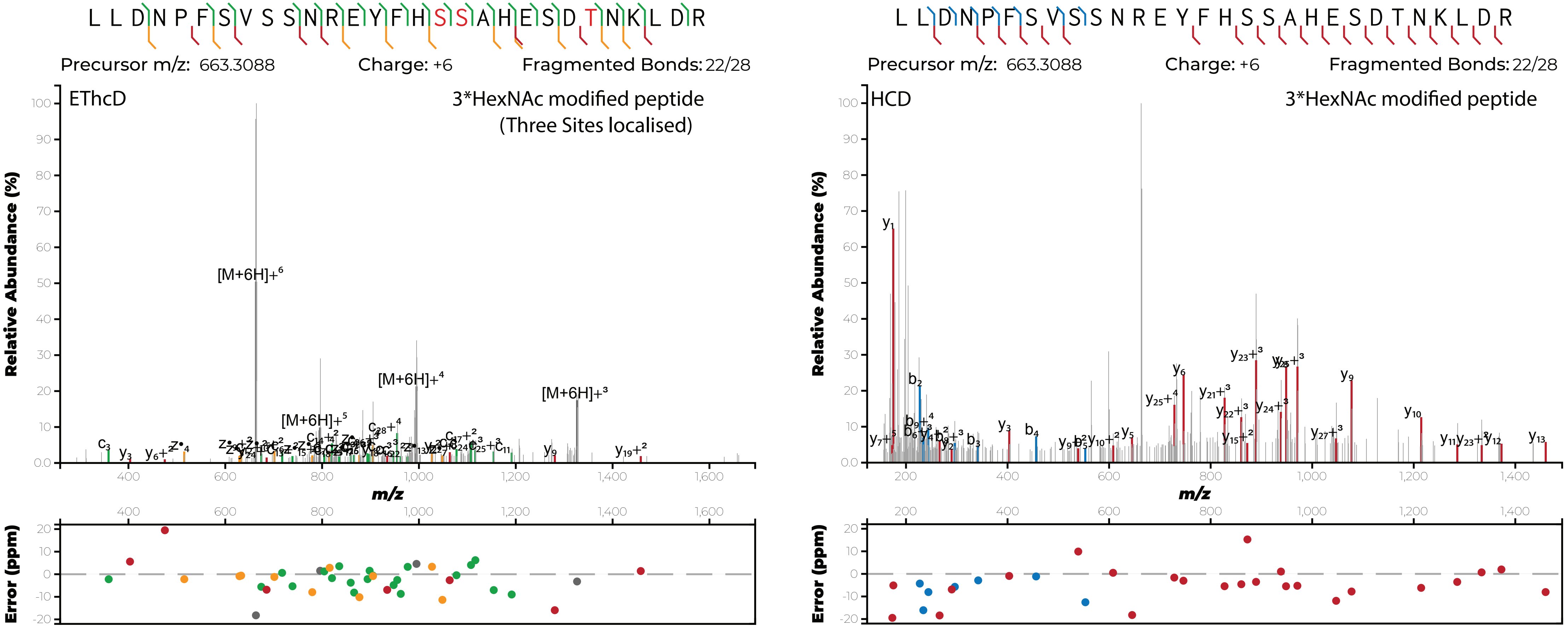

Q5CV53_CRYPI-Uncharacterized protein

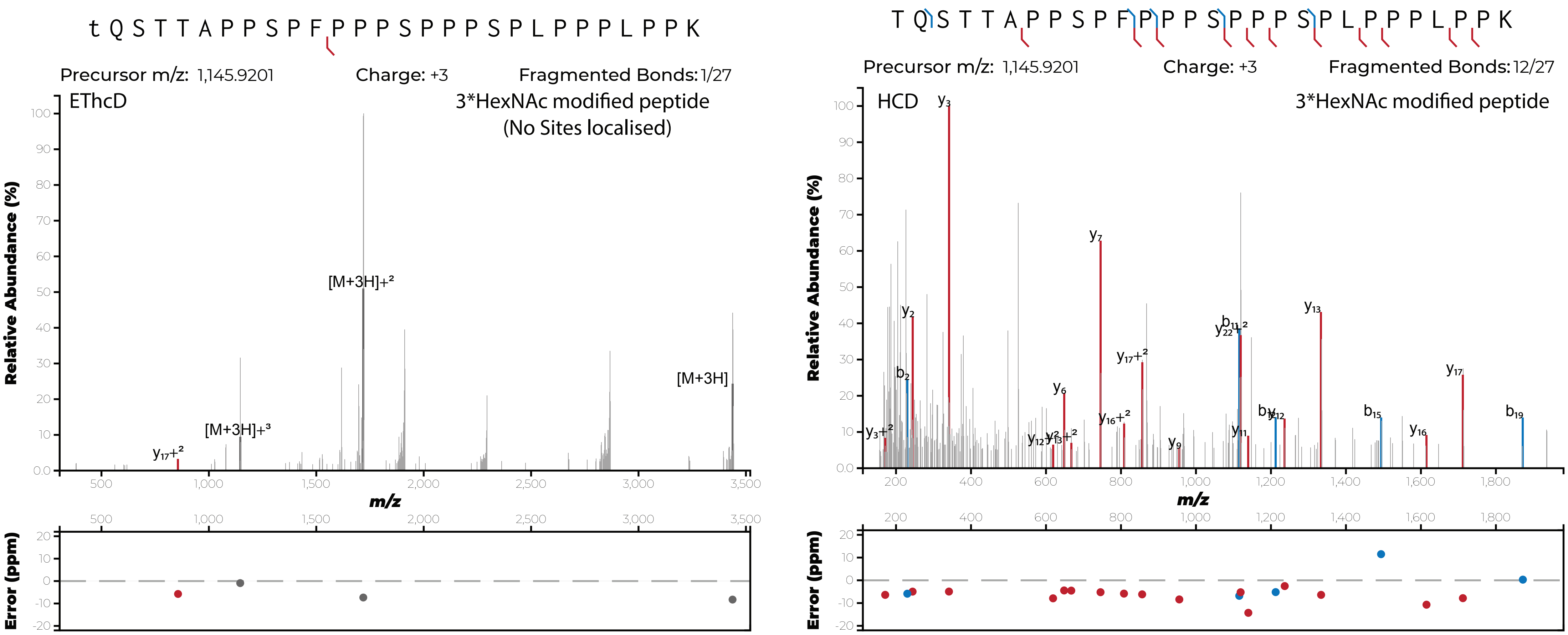

Q5CYR7_CRYPI-Uncharacterized protein

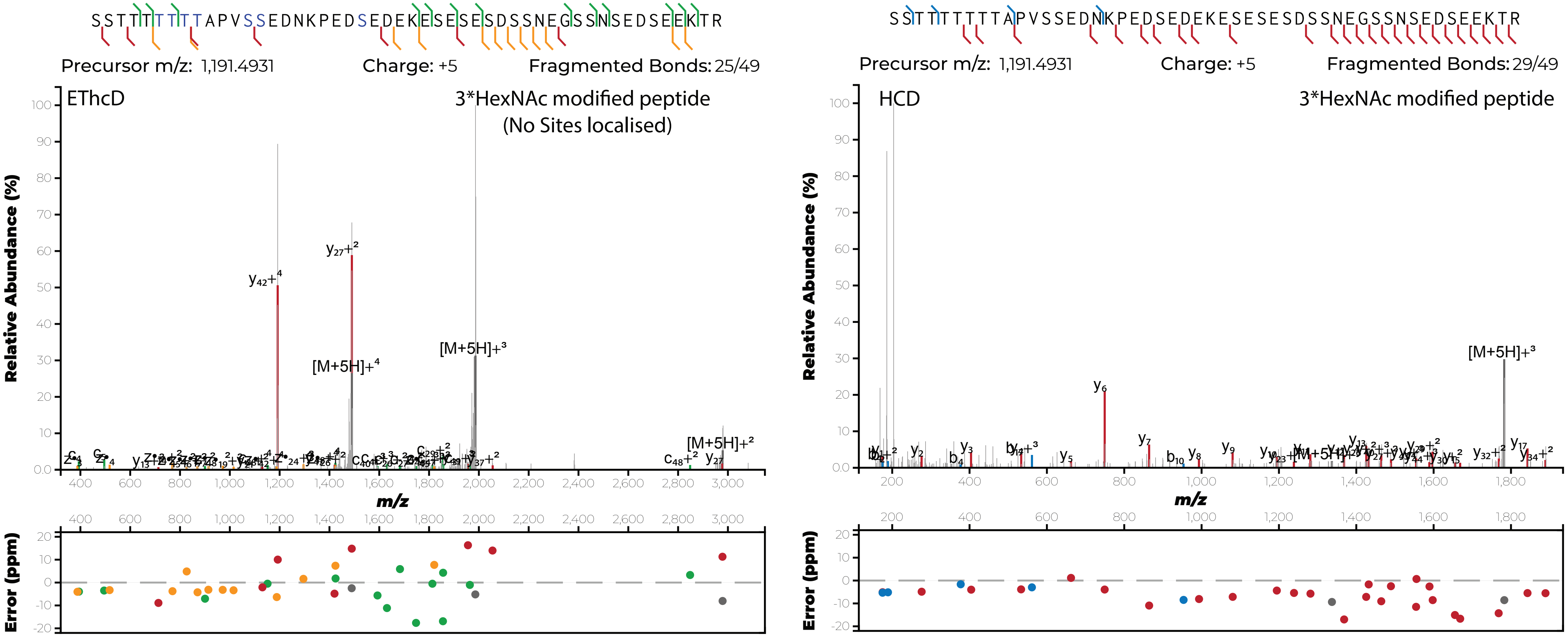

Supplement: Supplementary Data 1 [file mmc2.pdf]
